# Glycolysis/gluconeogenesis specialization in microbes is driven by biochemical constraints of flux sensing

**DOI:** 10.15252/msb.202110704

**Published:** 2022-01-07

**Authors:** Severin Josef Schink, Dimitris Christodoulou, Avik Mukherjee, Edward Athaide, Viktoria Brunner, Tobias Fuhrer, Gary Andrew Bradshaw, Uwe Sauer, Markus Basan

**Affiliations:** ^1^ Systems Biology Department Harvard Medical School Boston MA USA; ^2^ Institute of Molecular Systems Biology ETH Zurich Zurich Switzerland; ^3^ Applied Mathematics Department Harvard College Cambridge MA USA; ^4^ Laboratory of Systems Pharmacology Harvard Program in Therapeutic Science Harvard Medical School Boston MA USA

**Keywords:** flux sensing, lag time, metabolism, specialization, trade‐off, Metabolism, Microbiology, Virology & Host Pathogen Interaction

## Abstract

Central carbon metabolism is highly conserved across microbial species, but can catalyze very different pathways depending on the organism and their ecological niche. Here, we study the dynamic reorganization of central metabolism after switches between the two major opposing pathway configurations of central carbon metabolism, glycolysis, and gluconeogenesis in *Escherichia coli*, *Pseudomonas aeruginosa*, and *Pseudomonas putida*. We combined growth dynamics and dynamic changes in intracellular metabolite levels with a coarse‐grained model that integrates fluxes, regulation, protein synthesis, and growth and uncovered fundamental limitations of the regulatory network: After nutrient shifts, metabolite concentrations collapse to their equilibrium, rendering the cell unable to sense which direction the flux is supposed to flow through the metabolic network. The cell can partially alleviate this by picking a preferred direction of regulation at the expense of increasing lag times in the opposite direction. Moreover, decreasing both lag times simultaneously comes at the cost of reduced growth rate or higher futile cycling between metabolic enzymes. These three trade‐offs can explain why microorganisms specialize for either glycolytic or gluconeogenic substrates and can help elucidate the complex growth patterns exhibited by different microbial species.

## Graphical abstract

Microbes face a series of fundamental trade‐offs that limit their ability to optimize simultaneously for both glycolytic and gluconeogenic growth.

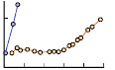

## Introduction

Whether in nature, microbiomes, or infections, microbes frequently encounter changing environments (Hardcastle & Mann, 1968; Fenchel, 2002; Stocker, 2012; Battin *et al*, 2016; Forsyth *et al*, 2018) and their ability to adapt quickly is a key determinant of fitness. But an understanding of the physiology of growth transitions, in particular what sets the time‐scales of adaptation, has remained largely elusive. For steady‐state exponential growth, metabolic models have made substantial progress over the last two decades, elucidating the flux and regulatory networks that govern the coordination of microbial metabolism (Bennett *et al*, 2009; Noor *et al*, 2010, 2014; Link *et al*, 2013; Bordbar *et al*, 2014; Chubukov *et al*, 2014; Gerosa *et al*, 2015; Vasilakou *et al*, 2016). Such metabolic models were successfully expanded to dynamic environments (Chassagnole *et al*, 2002; Chakrabarti *et al*, 2013; Zampar *et al*, 2013; Saa & Nielsen, 2015; Andreozzi *et al*, 2016; Yang *et al*, 2019) and used to gather kinetic information about metabolism, using perturbations (Link *et al*, 2013), stimulus response experiments (Chassagnole *et al*, 2002), or sequential nutrient depletion (Yang *et al*, 2019) to validate and improve metabolic models, but dynamic changes in metabolic shifts of growth conditions continue to pose a considerable challenge, and it is still unclear what determines how long bacteria need to adapt upon a change in the environment.

One example of such a switch happens when microbes deplete their primary nutrient. *Escherichia coli* preferentially utilizes hexose sugars such as glucose that are metabolized via glycolysis (Gerosa *et al*, 2015). To maximize growth on sugars, *E. coli* excretes substantial “overflow” production of acetate, even in the presence of oxygen (Basan *et al*, 2015a, 2017). This naturally leads to bi‐phasic growth, if no other microbe is around to utilize this bi‐product, where initial utilization of glucose is followed by a switch to acetate. Similar growth transitions from preferred glycolytic substrates to alcohols and organic acids ubiquitously occur for microbes in natural environments (Otterstedt *et al*, 2004; Buescher *et al*, 2012; Zampar *et al*, 2013). Since these fermentation products are all gluconeogenic, they require a reversal of the flux direction in the glycolysis pathway, which results in multi‐hour lag phases caused by the depletion of metabolite pools throughout the gluconeogenesis pathway (Basan *et al*, 2020). Similar long lag times in glycolytic to gluconeogenic shifts were observed for *Bacillus subtilis* and the yeast *Saccharomyces cerevisiae* (Basan *et al*, 2020). Shifts in the opposite direction, however, from gluconeogenic substrates to glycolytic ones, occur much more quickly in *E. coli* and other preferentially hexose fermenting microbes, in some cases even without detectable lag phases (Basan *et al*, 2020).

In our previous work (Basan *et al*, 2020), we showed how the growth rate dependence of enzyme expression leads to a universal relation between lag times and pre‐shift growth rates and found evidence that futile cycling at irreversible metabolic reactions plays an important role for causing lag times. However, we were unable to answer the most fundamental questions raised by these observations: Why are microorganisms such as *E. coli* or *S. cerevisiae* unable to overcome lag phases by expressing more metabolic enzymes or allosteric regulations that turn off futile cycling after metabolic shifts? Given the small number of enzymes involved in these irreversible reactions, their cost in terms of proteome allocation is likely minimal. Instead, microbes such as *E. coli* appear to be intentionally limiting enzyme expression and decreasing their growth rates on many glycolytic substrates (Basan *et al*, 2017). Moreover, why do shifts from glycolytic to gluconeogenic conditions result in lag times of many hours, while shifts from gluconeogenic to glycolytic conditions only take minutes? Given the symmetry of central metabolism, one would expect similar lag phases in the opposite direction. Is this preference for glycolysis a fundamental property of central metabolism or rather an evolutionary choice of individual species? At the core of these questions is a gap in understanding of how central carbon metabolism adjusts itself to nutritional changes.

Here, we study growth and metabolite dynamics of *E*. *coli*, *Pseudomonas aeruginosa*, and *Pseudomonas putida* using a kinetic model of central carbon metabolism to overcome this challenge. Our model coarse‐grains central metabolism to a low number of irreversible and reversible reactions, which allows us to focus on the dynamics of key metabolites and their regulatory action. The model couples metabolism to enzyme activity via allosteric regulation and enzyme expression to the concentration of regulatory metabolites via transcriptional regulation and flux‐dependent protein synthesis. Our formulation of metabolism and growth bridges fast metabolic time‐scales with slow protein synthesis. As we demonstrate, our model can explain major reorganizations of metabolism in response to nutrients shifts: the switching of the directionality of metabolic flux between glycolysis and gluconeogenesis. Dependent on the required directionality of flux in central metabolism, enzymes catalyzing the required flux direction are expressed and catalytically active, while enzymes catalyzing the opposite flux are expressed at low levels and their activities are repressed by allosteric regulation. This self‐organization is key for enabling fast growth and preventing costly futile cycling between metabolic reactions in opposing directions, which can inhibit flux and deplete ATP in the process.

Reestablishing this self‐organization after growth shifts is limited by biochemical constraints to sense fluxes and to regulate accordingly. When metabolite levels transiently collapse, allosteric and transcriptional regulation cannot distinguish between glycolysis and gluconeogenesis, rendering the cell unable to sense to the direction of flux. By choosing the activity of metabolic enzymes at these low metabolite levels to favor one direction, the cell can enable fast switching at the expense of the other direction. This choice of direction at low metabolite concentrations becomes the “default state” of central metabolism and determines the substrate preference.

According to the model, the preferred direction does not need to be glycolysis, and in principle gluconeogenic specialists with a gluconeogenic “default” state could have evolved, too. Indeed, we showed that *P*. *aeruginosa* shows reversed lag time, growth phenotypes and metabolite dynamics compared with those of *E. coli*, which verified that long lag times to glycolytic substrates are caused by the same inability to sense flux after nutrient shifts.

## Results

### An integrated, self‐consistent kinetic model of glycolysis / gluconeogenesis

In a shift between glycolysis and gluconeogenesis, flux in central metabolism needs to be reversed. To understand what limits the speed of adaptation between those two modes of flux, we turn to a theoretical model of central metabolism, but because the complexity of central metabolism with intertwined regulation at different levels prevents tracing quantitative phenotypes to their molecular origins, we sought to focus on the biochemical pathway topology with its key regulations that differentiate glycolysis and gluconeogenesis and constructed a minimal model of central metabolism. The model is illustrated in Box 1 and described in detail in the Appendix. It is based on topology of the biochemical network, the allosteric and the transcriptional regulation of the key the metabolic proteins of *E. coli*, all of which have been well characterized (Berger & Evans, 1991; Ramseier *et al*, 1995; Johnson & Reinhart, 1997; Pham & Reinhart, 2001; Kelley‐Loughnane *et al*, 2002; Hines *et al*, 2006; Fenton & Reinhart, 2009).

Box 1 **Integrated kinetic model of central carbon metabolism**

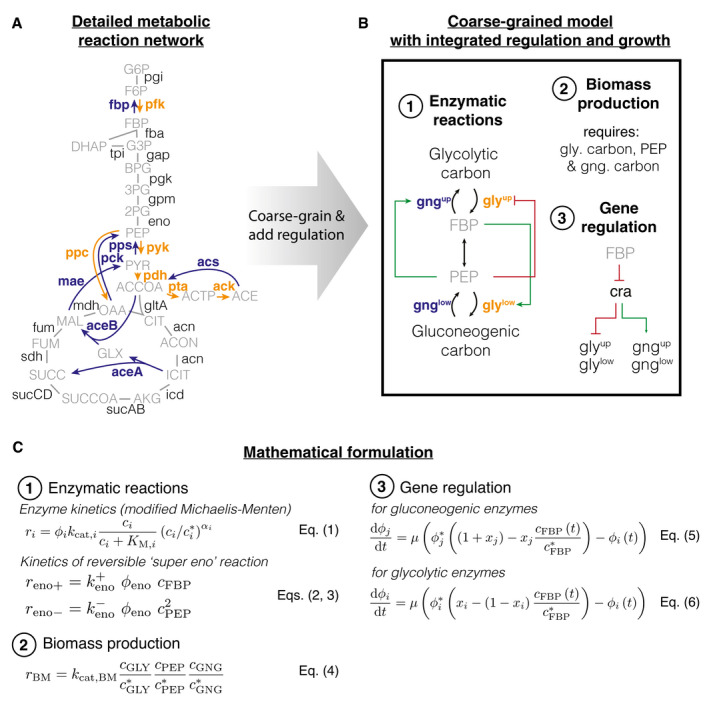

A, B (A) Detailed metabolic reaction network and (B) minimal network of central carbon metabolism. Coarse‐graining was done by combining irreversible glycolytic (orange) and gluconeogenic reactions (blue), as well as metabolites. Influx can either occur from glycolytic carbon sources (e.g., glucose) or gluconeogenic carbon sources, (e.g., tricarboxylic acid (TCA) cycle carbon‐like acetate or malate). (1) Gatekeepers to the central section of glycolysis and gluconeogenesis are the two irreversible reactions (gly^up^, gng^up^ and gly^low^, gng^low^) that feed and drain FBP and PEP. The irreversible reactions are allosterically regulated by FBP (fructose 1‐6‐bisphosphate) and PEP (phosphoenolpyruvate), where “outward” facing reactions are activated (green arrows) and “inward” facing reactions are repressed (red arrow). (2) Biomass production requires precursors from glycolytic carbons, PEP and gluconeogenic carbons (i.e., from TCA cycle). (3) Glycolytic and gluconeogenic enzymes are regulated by Cra, which is in turn modulated by FBP.
C Mathematical formulation of the model. Numbers correspond to features in panel (B). (1) Fluxes *r_i_* of enzymes *i* depend on enzyme abundances $\phi_{i}$, catalytic rates $k_{\text{cat},i}$ and allosteric regulations, modeled as a Hill function below its maximal saturation $({c_{j}/c_{j}^{*})}^{\alpha_{i}}$, where $c_{j}$ is the concentration of the regulatory metabolite and $c_{j}^{*}$ is a reference concentration. Reversible fluxes are modeled with simple mass action kinetics. (2) Biomass production is implemented in the model as single reaction that drains all three metabolites simultaneously at catalytic rate $k_{\text{cat,BM}}.$ (3) Enzyme expression depends linearly on FBP concentration $c_{\text{FBP}}$. Growth rate: *μ*, steady‐state abundance: $\phi_{i}^{*}$, steady‐state concentration $c_{\text{FBP}}^{*}$ and $x_{i}$ & $x_{j}$ modulate the sensitivity of regulation to FBP. Glycolytic and gluconeogenic enzymes are produced as part of protein synthesis. Thus in the model, flux through metabolism automatically leads to synthesis of metabolic enzymes and biomass production, resulting in dilution of existing enzymes.

The defining feature of the model is a coarse‐graining of the irreversible reactions (one‐directional arrows in “orange” and “blue”, Box 1A) in the upper and lower parts of central metabolism into single irreversible reactions (one‐directional “black” arrows in Box 1B). While not irreversible in an absolute sense, so‐called irreversible reactions are thermodynamically favored so much in one direction that they can be effectively considered as irreversible (Noor *et al*, 2014). As a result, these irreversible reactions in central metabolism need to be catalyzed by distinct enzymes that perform distinct reactions For example, fructose 6‐phosphate (F6P) is converted to fructose 1‐6‐bisphosphate (FBP) by enzyme PfkA using ATP. The opposite direction, FBP to F6P, is performed by a different enzyme, Fbp, which splits off a phosphate by hydrolysis. Each of the two reactions follows a free energy gradient and is irreversible. If both enzymes are present and active, then the metabolites will be continuously interconverted between F6P and FBP, and in each interconversion, one ATP is hydrolyzed to ADP and phosphate. This is a “futile cycle”. It drains the cell’s ATP resource and prevents flux going through the biochemical network. Because of this importance of irreversible reactions and futile cycling, we implement irreversible enzymes (“bold font, blue/orange” in Box 1A and B) and their allosteric regulation (“green” and “red” arrows in Box 1B) in the model. To successfully switch flux directions, the cell needs to express irreversible enzymes in the new direction, up‐regulate their activity, and repress enzyme activity in the opposing direction. Uptake of carbons from the environment is modeled as a flux to the substrates of the irreversible reactions, either glycolytic or gluconeogenic carbons depending on the availability.

The metabolites “sandwiched” between the irreversible reactions are coarse‐grained into the first and last metabolites of the series of reversible reactions, FBP and PEP (phosphoenolpyruvate). These metabolites regulate the activity and expression of the irreversible enzymes (Box 1B and Appendix Sec. 2).

In total, the model encompasses four irreversible reactions, each regulated allosterically by either FBP or PEP, and transcriptionally by FBP via Cra, and one reversible reaction that connects FBP and PEP. We used measured metabolite concentrations for growth on glucose (Kochanowski *et al*, 2013) and Michaelis constants (Berman & Cohn, 1970; Zheng & Kemp, 1995; Donahue *et al*, 2000) to constrain enzymatic parameters and biomass yield (Link *et al*, 2008) and density (Basan *et al*, 2015b) on glucose to constrain fluxes (Appendix Sec. 4). We used the level of futile cycling in the upper and lower reactions in exponential glucose growth, which summarize the effect of enzyme abundance and allosteric regulation, as fitting parameters such that the model reproduces the observed lag times in this paper; see Appendix Sec. 4.2 for details.

While the model in Box 1 was formulated to coarse‐grain glycolysis via the Embden–Meyerhof–Parnas (EMP) pathway, the dominant glycolytic pathway of *E. coli* growing on glucose (Gerosa *et al*, 2015), other glycolytic pathways, such as the Entner–Doudoroff (ED) or pentose phosphate pathway (PPP), have a similar topology. In ED glycolysis, phosphogluconate dehydratase (Edd) and KDPG aldolase (Eda) are irreversible reactions that feed into the chain of reversible reactions, analogous to 6‐phosphofructokinase (pfk) in the EMP pathway. The coarse‐grained model thus should capture these alternative pathways as well.

### Central carbon metabolism self‐organizes in response to substrate availability

**Figure 1:**
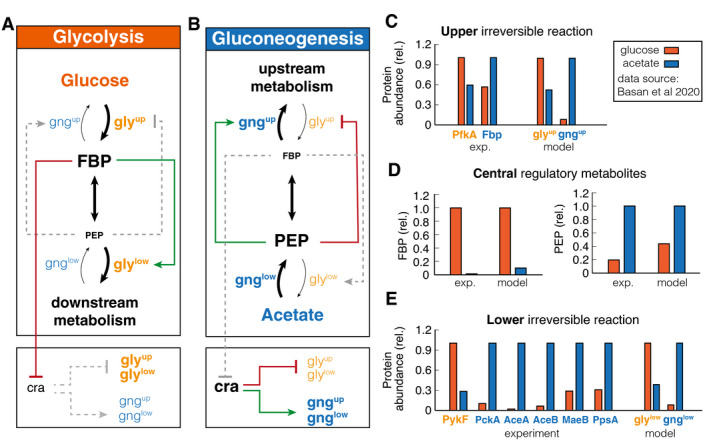
Self‐organization of metabolism in glycolysis and gluconeogenesis A, BGraphic summary of the reorganization in glycolysis and gluconeogenesis. Linewidth of reaction arrows indicates magnitude of flux. Font size of metabolites and enzymes indicates metabolite concentrations and enzyme abundances, respectively. Active regulation is indicated by red/green color, and inactive regulation is gray and dashed.C–ECalibration of model to experimental data (from Basan *et al*, 2020) metabolite concentrations and enzyme abundances relative to the highest concentration or abundance. Note the striking, differential regulation of FBP and PEP, high in one condition and low in the other.

To test whether this simple model could recapitulate steady‐state glycolytic and gluconeogenic growth conditions, we calibrated it with published metabolite and proteomics data of *E*. *coli*, which is well‐characterized in steady‐state exponential growth on glucose and acetate as sole carbon substrates (Basan *et al*, 2020). Indeed, the model reached distinct steady states for glycolytic and gluconeogenic conditions, which we summarized graphically with font size indicating enzyme and metabolite abundance and line widths indicating the magnitude of fluxes (Fig 1A and B). Active regulation is shown in colored lines, while inactive regulation is gray, dashed lines. We quantitatively compare enzyme and metabolite abundances to experimental measurements in Fig 1C–E and find that the coarse‐grained model can describe the reorganization of metabolism well, despite the simplifications of the metabolic and regulatory networks.

The simulation helps to understand how central metabolism self‐organizes in glycolytic and gluconeogenic conditions and how allosteric and transcriptional regulation optimize fluxes and minimize futile cycling during exponential growth. As shown in Fig 1C, in “orange”, during glycolytic conditions, the simulation reached a steady state with high FBP levels and low PEP levels. As illustrated in Fig 1A, the high FBP pool activates lower glycolysis, while the low PEP pool derepresses upper glycolysis and deactivates upper gluconeogenesis. This suppression of gluconeogenic fluxes in glycolysis reduces futile cycling, i.e., circular fluxes at the irreversible reactions, thereby streamlining metabolism. On a transcriptional level, the high FBP pool represses Cra, which in turn derepresses the expression of glycolytic enzymes and inhibits the expression of gluconeogenic enzymes. This results in high levels of glycolytic enzymes and low levels of gluconeogenic enzymes in the simulation (Fig 1D and E, right panels).

In gluconeogenic conditions (“blue” in Fig 1), we find precisely the complementary configuration of central carbon metabolism. Simulation and experiments show low FBP and high PEP pools (Fig 1C). As illustrated in Fig 1B, high PEP represses upper glycolysis and activates upper gluconeogenesis, while low FBP deactivates lower glycolysis. Low FBP also derepresses Cra, which leads to high expression of gluconeogenic enzymes and low expression of glycolytic enzymes (Fig 1D, right panels).

**Figure EV1:**
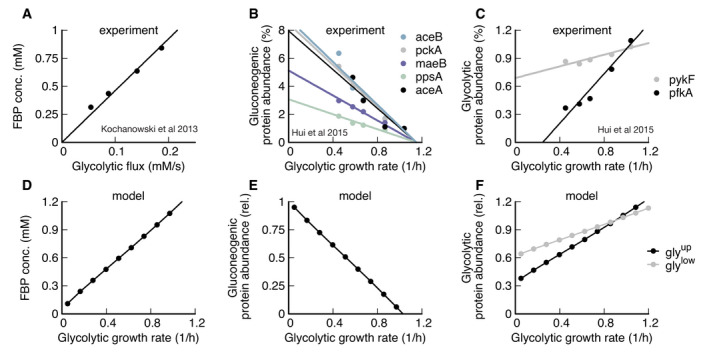
Metabolic state depends on growth rate ADuring glycolytic growth, FBP linearly increases with growth rate (Kochanowski *et al*, 2013).BGluconeogenic enzymes decrease linearly with glycolytic growth rate (Hui *et al*, 2015).CGlycolytic enzymes increase linearly with glycolytic growth rate (Hui *et al*, 2015).D–FSimulation results recapitulate experimental evidence.

Next we tested if the model could recapitulate how varying growth rates on glycolytic and gluconeogenic nutrients affects metabolite levels and protein expression in *E. coli* (Gerosa *et al*, 2015; Hui *et al*, 2015). In particular, it has been shown experimentally that FBP acts like a flux sensor and FBP concentration linearly increases with glycolytic flux (Fig EV1A) (Kochanowski *et al*, 2013), which is recapitulated by our simulation (Fig EV1D), under the condition that enzymes catalyzing the reversible reaction are far from saturation. The linear increase in FBP concentration with growth rate results in a linear growth rate dependence of gluconeogenic and glycolytic enzyme abundances in the simulation, in good agreement with experimental measurements of enzyme abundances from proteomics (Fig EV1 compare B and C with E and F) (Hui *et al*, 2015). Together, these results show that integrating the transcriptional and allosteric regulation of FBP and PEP in the coarse‐grained model suffices to describe the major re‐configuration of central metabolism in glycolysis and gluconeogenesis.

### Central carbon metabolism of *E. coli* is primed for switches to glycolysis

**Figure 2:**
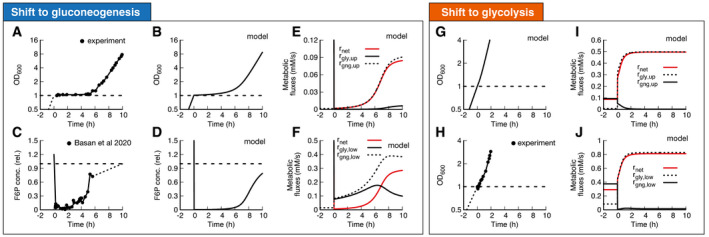
Shifts between glycolysis and gluconeogenesis A, B(A) Experimental and (B) model of optical density after shift of *E. coli* from glucose to acetate. Growth shows a substantial lag before it recovers.C, D(C) Experimental and (D) model of F6P (normalized to the final state) collapses after shit to acetate and continues to stay low throughout lag phase. Because F6P is an essential precursor for biomass production, this limitation effectively stops biomass growth. Data points show a single time‐series from (Basan *et al*, 2020).E, FFluxes of all irreversible reactions in units of intracellular concentration per time. Especially fluxes in lower glycolysis/gluconeogenesis are of equal magnitude, leading to a futile cycle, where no net flux (red line) through central carbon metabolism can be established.G–JOptical density and metabolic fluxes for the reversed shift from acetate to glucose show immediate growth and no intermittent futile cycling. The dynamics of all enzyme abundances, regulation, and fluxes for both shifts are shown in Appendix Figs S1–S5 in detail. The model also correctly predicts that enzyme abundances only adapt late in the lag phase (Appendix Fig S6).

Equipped with this model, we next address the mechanistic basis for the extended lag phases of *E. coli* upon nutrient shifts from glycolytic to gluconeogenic conditions. When shifted from glucose to acetate *E. coli* shows a lag time with almost no growth for around 5 h (Fig 2A, data: (Basan *et al*, 2020)). We can reproduce this lag with our model (Fig 2B, Appendix Figs S1–S5) when we fit pre‐shift futile cycling, which is a measure for enzyme abundances and allosteric regulations; see Appendix Sec. 2 for details. All model solutions for *E. coli* shown in this paper are generated with the parameters generated from this fit. The model captures the slow adaptation of glycolytic and gluconeogenic enzymes, the major change of which occurs only toward the end of the lag phase (Appendix Fig S6). Investigating the origin of the growth arrest in the simulation, we found that during lag phase, the concentrations of upper glycolytic precursors (which includes fructose 6‐phosphate (F6P) and glucose‐6‐phosphate (G6P)) remained very low compared with their steady‐state values, which matches published experimental evidence of F6P measurements (Basan *et al*, 2020) (Fig simulation: 2C, data 2D). This indicates that essential precursors are limited, and thereby, according to equation (4) growth rate during lag phase stalls.

**Figure 3:**
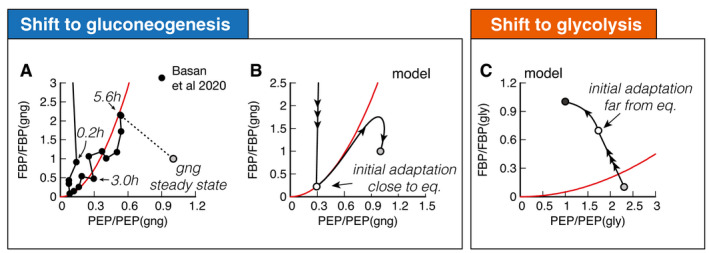
Molecular cause for asymmetric recovery dynamics in *E. coli* Recovery of FBP and PEP of after a shift from glucose to acetate shows a distinctive joint increase, followed by an overshoot of FBP. Red line is a quadratic guide to the eye. Final acetate steady state is drawn as gray symbol and used to normalize both FBP and PEP levels. Data are a single time‐series from (Basan *et al*, 2020).Model solution of FBP and PEP. After the fast collapse of metabolite levels (triple arrow to white circle), the dynamics closely follows the quadratic FBP‐PEP equilibrium equation (7). Eventually recovery will diverge away from the equilibrium line, toward the non‐equilibrium steady states of gluconeogenesis (gray circle).For a shift to glycolysis, metabolite levels do not collapse, but instead land already far from equilibrium (triple arrow to white circle), such that flux is immediately established, and recovery is quick.

In the simulation, the F6P limitation is caused by low net fluxes in upper and lower gluconeogeneses (Fig 2E and F, red lines). Previously, it was suggested that futile cycling between gluconeogenic and glycolytic enzymes could contribute to this flux limitation (Basan *et al*, 2020), supported by the observation that overexpression of glycolytic enzymes in upper or lower glycolysis strongly impaired switching and resulted in much longer lag times (Basan *et al*, 2020). The simulation allows us to probe the effect of futile cycling *in silico*, which cannot be directly measured experimentally. Indeed, we found for our default *E. coli* parameters that residual lower glycolytic flux almost completely canceled the flux from gluconeogenesis, i.e., $r_{\mathrm{gly}}^{\mathrm{low}}\approx r_{\mathrm{gng}}^{\mathrm{low}}$ (solid and dashed black lines in Fig 3F), such that net gluconeogenic flux remained close to zero (red line, Fig 2E and F). Thus, this futile cycling appears to be the main reason for limiting net flux throughout the lag phase.

The biochemical network and regulation are almost completely symmetric with respect to the direction of flux, so one might naively expect a shift from gluconeogenesis to glycolysis to also result in a long lag. However, experimentally the shift in the opposite direction from gluconeogenesis to glycolysis occurs very quickly in *E. coli* (Fig 2G) (Basan *et al*, 2020). Our simulations with the standard *E. coli* parameters can recapitulate that central metabolism adjusted very quickly and growth resumed without a substantial lag phase (Fig 2H). In striking contrast to the shift to gluconeogenesis, futile cycling played no role in the shift to glycolysis, because both upper and lower glycolytic fluxes got repressed immediately after the shift (Fig 2I and J, solid black line), such that net flux could build up (Fig 2I and J, red line). The absence of transient futile cycling, despite the symmetry of regulation and metabolic reactions, means that, according to the model, it must be the allosteric and transcriptional regulations that “prime” central metabolism of *E. coli* for the glycolytic direction.

### Molecular cause of preferential directionality

To understand the molecular cause of the asymmetric response and lag phases, we investigated the role of allosteric and transcriptional regulation in our simulation. During steady‐state growth, the differential regulation during glycolysis and gluconeogenesis is achieved by PEP and FBP, the metabolites that are “sandwiched” between the two irreversible reactions and connected by a series of reversible enzymes, coarse‐grained in our model into the “super‐eno” enzyme. First, we focused on regulation during exponential growth and wanted to investigate how the cell achieves differential regulation of glycolytic and gluconeogenic enzymes using the metabolites FBP and PEP. In equilibrium, forward and backward reactions would balance, i.e., $r_{\mathrm{ENO}+}=r_{\mathrm{ENO}‐}$, and no net flux could run through central metabolism, meaning that the cell could not grow. Using equations (2 and 3), the balance of forward and backward fluxes results in a fixed quadratic dependence of FBP and PEP in equilibrium,

(1) $$c_{\text{FBP}}^{\text{eq}}=k_{\text{ENO}‐}/k_{\text{ENO}+}{\left(c_{\text{PEP}}^{\text{eq}}\right)}^{2}.$$

The form of equation (1) is specific to F6P converting to FBP being the irreversible step of upper glycolysis and can change if pathways such as Entner–Doudoroff (ED) or pentose phosphate pathway (PPP) are dominant.

Close to the equilibrium, FBP and PEP levels go up and down together, rather than the opposing directions, as observed for glycolytic and gluconeogenic growth (Fig 1A and B). This results in low net flux and very slow growth. Hence, for steady‐state growth, the equilibrium must be broken and FBP≫PEP or FBP≪PEP, such that either glycolytic flux is bigger than gluconeogenic or vice versa ($r_{\mathrm{ENO}+}\;\gg \;r_{\mathrm{ENO}‐}$ and $r_{\mathrm{ENO}+}\;\ll \;r_{\mathrm{ENO}‐}$, respectively). This is achieved by the irreversible reactions, which drain and supply metabolites to the “super‐eno”. Because of the positive feedback between enzyme activity and non‐equilibrium of the “super‐eno”, this regulation topology achieves differential regulation during glycolysis and gluconeogenesis. As we observed in the analysis of the glycolytic and gluconeogenic steady states (Fig 1), this differential regulation adjusts enzyme levels via transcriptional regulation and suppresses futile cycling at the irreversible reactions.

While regulation of central metabolism efficiently organizes FBP‐PEP in a far‐from‐equilibrium state during exponential growth, nutrient shifts expose the limitations of this regulatory system. To understand why, we plot FBP against PEP, with both metabolites normalized to their gluconeogenic steady state (Fig 3A). We indicated several time‐points along the dynamics, and the final steady state is shown with a gray symbol. Initially, both FBP and PEP drop close to zero, followed by a very slow joint increase in FBP and PEP over the course of hours (Fig 3A). This joint increase, rather than a differential increase, is the hallmark of a close‐to‐equilibrium state.

The slow recovery can be understood from the simulation, which shows that FBP and PEP proceed close to the equilibrium line of equation (7), where growth is slow (Fig 3B). As a guide to the eye, we drew an equilibrium parabola in Fig 3A along the joint increase, too.

Higher gluconeogenic flux from increasing levels of gluconeogenic enzymes is almost completely lost to a corresponding increase in futile cycling because increasing FBP activates lower glycolysis, instead of deactivating it (Fig 2F). The overshoot of FBP in Fig 3A (data) and Fig 3B (model) is what finally allows the cell to establish net flux because it is breaking the equilibrium: PEP concentration is high enough to activate upper gluconeogenesis sufficiently to drain FBP via upper gluconeogenesis (see Fig 2E). Lower FBP then shuts down futile cycling in lower glycolysis/gluconeogenesis (Fig 2F), pushing FBP and PEP concentrations to a state far from the equilibrium line (see Fig 3B) and allowing the cell to grow at a faster rate.

The fundamental difference between shifts to gluconeogenesis and glycolysis in *E. coli* is that glycolytic shifts immediately land far from equilibrium (Fig 3C, triple arrow to white circle), such that cells immediately grow at faster rates, allowing them to express the new enzymes needed to recover quickly. But why does one direction immediately land far from equilibrium, while the other lands close to equilibrium?

### Three trade‐offs constrain lag times to glycolysis and gluconeogenesis

**Figure 4:**
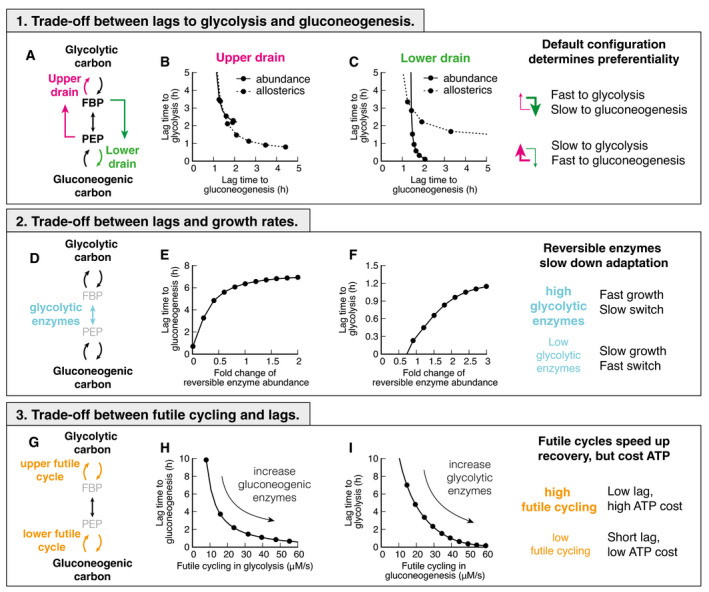
Trade‐offs between glycolysis and gluconeogenesis ATwo drains in central metabolism deplete central metabolites.B, CChanging abundance ϕ or allosteric regulation strength α in either lower or upper drain leads to a shift of lag times, decreasing lags in one direction at the cost of the other. Chosing strength of the drains such that either top or bottom is stronger, will lead to a fast recovery in on direction, and a slow in the other.DReversible enzymes in the central metabolism (coarse‐grained here into “super‐eno”). Abundance of reversible enzymes scale linearly with growth rate [16].E, FDecreasing abundance of reversible enzymes decreases lag times. This effect is due to regulatory metabolites being in a far‐from‐equilibrium state when abundances are low, which allows differential regulation via FBP and PEP. For high abundance, regulation is weak and lag times long.GThere are two futile cycles in central metabolism.H, IIncreasing abundance of enzymes of the opposing direction in pre‐shift, e.g., gluconeogenic enzymes in glycolytic growth, increases futile cycling and decreases lag times. Because in futile cycles free energy is dissipated, usually in the form of ATP hydrolysis, futile cycling has an energetic cost.

The out‐of‐equilibrium state is caused by net flux going through metabolism. Therefore, we investigated what causes fluxes not to flow in a uniform direction after shifts to glycolysis and gluconeogenesis. In principle, metabolite flux brought to the “super‐eno” can exit via two drains: upper gluconeogenesis, activated by PEP, and lower glycolysis, activated by FBP (Fig 4A). How much flux exits via either drain depends on the current protein abundances and the allosteric regulation. If the allosteric regulation and protein abundances favor the lower drain, then after a switch to glycolysis, FBP builds up, PEP is drained, and a net flux is immediately accomplished. In a shift to gluconeogenesis, however, flux that enters central metabolism from the TCA cycle will immediately drain back to the TCA cycle, leading to an in‐and‐out flux but no net flux. In this situation, FBP and PEP stay in equilibrium and the recovery stalls. If on the other hand, the upper drain was favored over the lower drain, then we would expect the behavior to be reversed and gluconeogenic flux would be immediately accomplished, while the glycolytic recovery would stall.

In the simulation, we are able to test the hypothesis that the upper and lower drains determine the preferential directionality of the central metabolism by varying enzyme abundances and the strength of allosteric interactions in upper and lower drains *in silico*. We let metabolism adapt to gluconeogenic and glycolytic conditions and calculate lag times (Fig 4B and C). Indeed, we found that a decrease in lag time in one direction led to an increase in lag time in the opposite direction.

Varying the outflow from metabolism is not the only determinant of lag times. The set of reversible enzymes, coarse‐grained in our model into the “super‐eno”, plays another key role, because it interconverts the regulatory metabolites FBP and PEP (Fig 4D). If this conversion is fast, the concentrations of FBP and PEP will be close to their equilibrium relation in equation (7), and differential regulation will be impossible. As a result, lag times in both directions increase if we increase the abundance of reversible reactions (Fig 4E and F). This is a counter‐intuitive result, as one would naïvely expect more enzymes to speed up reactions, but instead, more enzymes collapse the differential regulation and slow down adaptation rates. This trade‐off is unavoidable for fast‐growing cells because the cell needs a sufficient amount of reversible glycolytic enzymes to catalyze metabolic flux.

Finally, lag times depend on the amount of futile cycling, i.e., the circular conversion of metabolites in the upper and lower irreversible reactions (Fig 4G). Increasing the abundance of gluconeogenic enzymes in glycolytic growth or glycolytic enzymes in gluconeogenic growth increases futile cycling, but decreases lag times (Fig 4I and H). Because futile cycling dissipates ATP, which is not explicitly built into our model, this third trade‐off means that organisms can decrease their switching times by sacrificing energetic efficiency.

**Figure 5:**
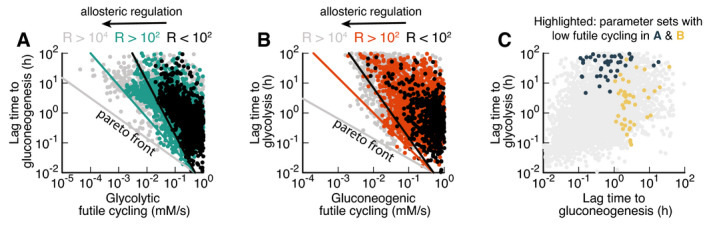
Large‐scale parameter scan reveals Pareto optimality between lag times and futile cycling A, BModel calculated for randomized sets of protein abundancies, reaction rates, Michaelis constants, allosteric interactions, transcriptional regulation, see Appendix. Each point corresponds to a parameter set that allows exponential growth on both glycolytic and gluconeogenic carbons, as well switching between both conditions. Data are colored according to the total regulation R, i.e., the sum of fold changes in enzyme activities between glycolysis and gluconeogenesis, ${\left(c_{i}^{\mathrm{gly}}/c_{i}^{\mathrm{gng}}\right)}^{\alpha_{i}}$, where $c_{i}^{\mathrm{gly}}$ and $c_{i}^{\mathrm{gng}}$ are protein abundances in glycolysis and gluconeogenesis of protein i and $\alpha_{i}$ the strength of the allosteric regulation. For standard E. coli parameters R = 23. R > 10^4^ are likely unphysiological. Lines indicate Pareto front and are drawn by hand.CParameter sets from panels (A) and (B) with low futile cycling highlighted over the background of all parameter sets (gray).

Are these three trade‐offs a fundamental consequence of the regulatory structure or are there parameter combinations that avoid the trade‐offs by simultaneously enabling rapid growth and rapid switching without costly futile cycling? To answer this question, we performed an extensive scan of model parameters by randomly choosing sets of biochemical parameters and simulating the resulting model. Of those parameter sets, we chose those that allowed steady‐state growth in both glycolytic and gluconeogenic conditions and were able to switch between both states. We plotted the sum of futile cycling in the upper and lower irreversible reactions in the pre‐shift conditions against the subsequent lag times for shifts to gluconeogenesis (Fig 5A) and to glycolysis (Fig 5B). In addition, we colored individual parameter sets according to the total allosteric regulation, defined as the sum of fold changes in enzyme activities between glycolysis and gluconeogenesis (black: R < 10^2^, red/green: 10^4^ > R > 10^2^, gray: R > 10^4^). These fold changes are the result of both allosteric and transcriptional variations. We found that metabolism in the majority of randomly generated models is inefficient and dominated by futile cycling; only a minority of models were able to reduce futile cycling in glycolysis and gluconeogenesis. Remarkably, despite probing variations of all possible model parameters, including Michaelis Menten parameters of enzymes and the strengths of allosteric and transcriptional regulation, lag times could not be reduced at‐will by the cell. Instead, individual parameter sets with similar allosteric regulation (colors) are bound by a “Pareto frontier” (solid lines) between futile cycling in pre‐shift conditions and lag times. Points close to the “Pareto frontier” are Pareto‐optimal, meaning that any further decrease of either parameter must come at the expense of the other. Overall, stronger allosteric regulation shifted the Pareto frontier, but was not able to overcome it. Parameter combinations that led to low futile cycling in either glycolysis or gluconeogenesis showed long lag times in at least one condition (Fig 5C, “black” and “yellow”) compared with the background of all simulated parameter sets (“gray”). Thus, from this analysis, it seems that organisms with the regulatory architecture of Box 1 cannot overcome long lag times without paying a futile cycling cost during steady‐state growth.

### Gluconeogenesis specialists are constrained by the same trade‐offs

Taken together, the results of Figs 4 and 5 suggest that microbial cells cannot achieve fast growth, low futile cycling, and fast adaptation simultaneously in both glycolysis and gluconeogenesis. Instead, trade‐offs between these six objectives constrain the evolutionary optimization of microbial metabolism, such that any optimal solution is on a surface of a multidimensional Pareto frontier, where any improvement in one phenotype will come at the expense of others. To test this hypothesis, we next asked whether a gluconeogenic specialist would indeed be constrained by the same trade‐offs as *E. coli* and other glycolytic specialists. For this purpose, we chose *P*. *aeruginosa,* a well‐studied gluconeogenesis specialist that has a similar maximal growth rate in minimal medium as *E. coli* (*E. coli* 0.9/h on glucose, *P. aeruginosa* 1.0/h on malate) and grows on a wide variety of substrates.

**Figure 6:**
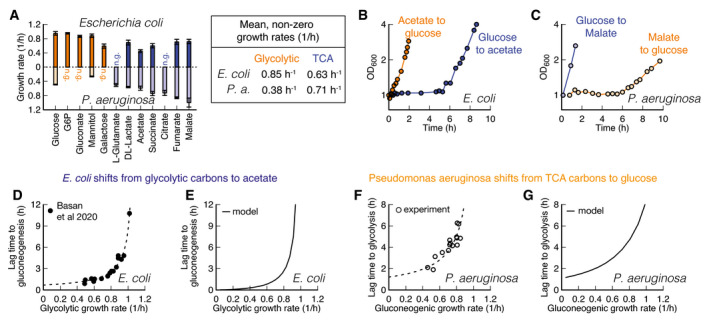
Comparison of *E. coli* and *P. aeruginosa* during growth and shifts AGrowth rates on glycolytic carbons (orange) are faster for *E. coli* than on gluconeogenic carbons (blue). For *Pseudomonas*, this dependence is reversed. No growth indicated with “n.g”. Data are average of *n* = 3 biological replicates. Error bar is standard deviation.B, CShifts for *E. coli* and *P. aeruginosa* between glycolytic and gluconeogenic carbon substrates. The preferential order of *P. aeruginosa* is reversed in comparison with *E. coli*.D
*E. coli* shows an increase in lag times to gluconeogenesis with increasing pre‐shift growth rate. Lag times diverge around growth rate 1.1/h. Each point is an individual experiment.EThe model predicts diverging growth rates without further fitting, based on the growth rate‐dependent expression levels of glycolytic and gluconeogenic enzymes (Fig 2E and F).F
*P. aeruginosa* shows a strikingly similar growth rate to lag time dependence as *E. coli*, when switched to glycolysis, with lag times diverging around 1.0/h. Each point is an individual experiment.GThe model can recapitulate observed *P. aeruginosa* lag times if pre‐shift glycolytic enzymes are decreased as a function of pre‐shift growth rate.

Strikingly, *P. aeruginosa* grows fast on gluconeogenic substrates that are considered “poor” substrates for *E. coli*, but slow on glycolytic substrates that are considered “good” (Fig 6A). From our model, we would expect that such a specialization for gluconeogenic substrates would go along with a reversal in lag phases, too. Indeed, switching between glycolytic and gluconeogenic substrates, *P*. *aeruginosa* exhibits a mirrored pattern of lag phases compared to *E. coli* (compare Fig 6B and C), with a long multi‐hour lag phase when switched to glycolysis.

To investigate whether both *E. coli* and *P. aeruginosa* are constrained by the same trade‐offs, we investigated the effect of pre‐shift growth rate, which according to Fig 4 should have a negative effect on growth rates. For *E. coli,* it is known that shifts from glycolysis to gluconeogenesis depend on the pre‐shift growth rate (Fig 6D, data: (Basan *et al*, 2020)), which we can capture in our model if we take FBP‐dependent transcriptional regulation into account (Fig 6E). We tested the corresponding lag times for *P. aeruginosa* by varying gluconeogenic substrates and found a similar dependency in shifts to glycolytic substrates (Fig 6F and G). Hence as expected from the model, these findings show that *P*. *aeruginosa* is exhibts the same trade‐offs as *E. coli*.

To decipher whether *P. aeruginosa* lag times are constrained on a molecular level by the same inability to break the equilibrium after nutrient shifts, we investigated metabolite concentration dynamics in central metabolism. Because *P*. *aeruginosa* uses the ED pathway for hexose catabolism (Wang *et al*, 1959; Vicente & Cánovas, 1973), we needed to adapt our model slightly. The irreversible reactions in the ED pathway convert gluconate‐6‐phosphate to glyceraldehyde 3‐phosphate (GAP) and pyruvate. In the reversible chain of reactions, the first metabolite in glycolysis is thus GAP rather than FBP. Because GAP is difficult to quantify in mass spec‐based metabolomics, we used the closely related compound dihydroxyacetone phosphate (DHAP) as a proxy. DHAP is in chemical equilibrium with GAP via a single fast and reversible isomerase (Nikel *et al*, 2015).

**Figure 7:**
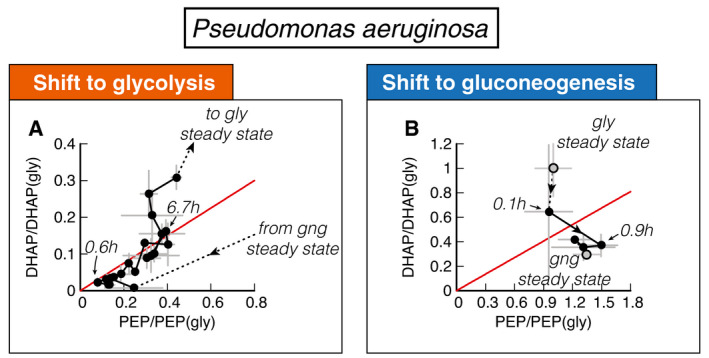
Metabolite dynamics of *P. aeruginosa* during shifts from malate to glucose and vice versa DHAP and PEP during shift from malate (“gng”) to glucose (“gly”), normalized to the final glycolytic steady state. Recovery follows a direct proportionality, indicating that central metabolism is close to equilibrium (red line) during the recovery.DHAP and PEP reach the final steady state (“gng”) without creeping along the equilibrium line. Data information: Data are the average of *n* = 3 biological replicates, and error bars show standard deviation.

Analogous to Fig 3, we plot the dynamics of DHAP versus PEP, normalized to their glycolytic steady‐state values, for both shifts (Fig 7A and B). The dynamics starts and ends at their respective steady states (gray symbols and dashed lines) and follows the direction of the indicated arrow. In the chemical equilibrium, DHAP depends linearly on PEP, $c_{\mathrm{DHAP}}^{\mathrm{eq}}=k_{\mathrm{ENO}‐}/k_{\mathrm{ENO}+}\;c_{\mathrm{PEP}}^{\mathrm{eq}},$ analogous to equation (7), but without the square because of the 1‐to‐1 stoichiometry between DHAP and PEP. This equilibrium is indicated with a red line. During the long lag time of *P. aeruginosa* in a shift from malate to glucose, we see that initially both DHAP and PEP collapse, followed by a slow increase along the equilibrium line (Fig 7A). Thus, despite substantial amounts of metabolites being built‐up, “super‐eno” remains close to equilibrium. Only after 5.6 h, when the DHAP‐PEP dynamics deviates from the line, the equilibrium is broken and net flux can be achieved.

In the reverse shift from glucose to malate, *P. aeruginosa*, in contrast, can immediately establish a non‐equilibrium and grow. Thus not only is the asymmetry in lag times reversed compared to *E. coli*, it is also caused by the same inability to break the equilibrium and establish net flux in central metabolism.

**Figure EV2:**
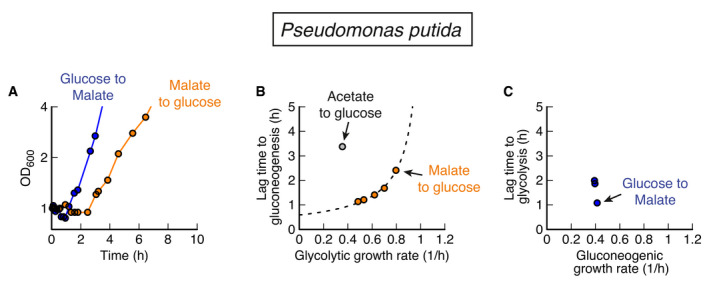
*Pseudomonas putida* shifts between glycolytic and gluconeogenic carbon substrates For shifts between glucose and malate, and vice versa, P. putida shows moderate lag times in both directions.Lag times depend on pre‐shift growth rate for glycolysis to gluconeogenesis, with acetate to glucose being a clear outlier.Lag times for gluconeogenic to glycolytic shifts are of the same magnitude as for the reverse direction. Compared to *P. aeruginosa*, *P. putida* shows longer lags from glucose to malate (0.55 h compared to 1.1 h) and slower growth on the fastest carbon (malate) (1.0/h compared to 0.8/h).

But, microbes do not have to be optimized for either direction. One such case is *P. putida* with moderate lag times of about 1 to 2 h in both directions and only a slight preference for gluconeogenic substrates (Fig EV2). According to the model, such a generalist strategy can also be a Pareto‐optimal solution of the biochemical trade‐offs of Figs 4 and 5, but it must come at the expense of no fast recovery (Fig 4A–C) and reduced growth, because of the trade‐offs with reversible enzymes (Fig 4D–F) and futile cycling (Fig 4G–I). This is indeed the case for *P. putida*. Lag times are in the fast direction twice as long compared with *P. aeruginosa,* and the growth rate is about 20% slower (Fig S8).

## Discussion

In this work, we presented a coarse‐grained kinetic model of central carbon metabolism, combining key allosteric and transcriptional regulation, as well as biomass production, enzyme synthesis, and growth. This model elucidates the remarkable capacity of central carbon metabolism to self‐organize in response to substrate availability and flux requirements. During exponential growth, regulatory metabolites adjust to far‐from‐equilibrium steady states, providing the cell with an elegant mechanism to sense the required directionality of the flux, but the model reveals a key limitation of this flux sensing. Because after a nutrient shift the concentration of the metabolites collapses to its equilibrium, the cell becomes “blind” to the direction that the flux is supposed to flow through the system. By implementing a preferred direction, the cell can partially overcome lag times in one direction at the cost of increasing lag times in the opposite direction. In addition, two more trade‐offs constrain the ability to simultaneously decrease both lag times, because it impacts growth rate and the level of futile cycling during growth.

Microbial species can maximize their proliferation only up to the Pareto frontier spanned by these trade‐offs, which can lead to evolution of substrate specialization. We validated this key model prediction in different bacterial species. In *P. aeruginosa,* we showed a reversal of substrate preference as compared to *E. coli*, which coincided with a complete reversal of the phenomenology of lag phases and metabolite dynamics. In *P. putida,* we found a generalist strategy with moderate lag times in both directions.

One of the results from our model is that lag times could be substantially reduced by allowing futile cycling, e.g., by expressing irreversible enzymes for both directions at all times. The proteome cost of such a wasteful strategy would be relatively low. Because energy production pathways only constitute a relatively small fraction (around 20% (Basan *et al*, 2015c)) of the total cellular proteome and nutrient uptake even smaller (around 1% for glucose uptake (Schmidt *et al*, 2016)), the cell could compensate ATP dissipated in futile cycling by increasing nutrient uptake and ATP production at a relatively low proteome cost. However, experimentally it appears that *E. coli* chooses to keep futile cycling in check by transcriptionally regulating irreversible enzymes. We thus hypothesize that the cost of futile cycling must be considered in conditions where the energy budget is much more limited, such as growth shifts and during starvation. In fact, it has been recently shown that the energy budget of the cell is around 100‐fold smaller during carbon starvation and that energy dissipation can increase death rates several fold (Schink *et al*, 2019). Therefore, even levels of futile cycling that are modest during steady‐state growth should severely affect survival of cells in these conditions.

Our findings indicate that the identified trade‐offs are inherent properties of central carbon metabolism, at least given the existing allosteric and transcriptional regulation. But could different regulation overcome this limitation? In theory, the cell could use a direct input signal from the carbon substrate to allosterically inhibit or even degrade undesired metabolic enzymes. This would uncouple enzyme abundances and activities in pre‐ and post‐shift growth and circumvent the trade‐offs, but with dozens of glycolytic and gluconeogenic substrates, this would result in a much higher degree of regulatory complexity, potentially exceeding the regulatory signal capacity that microbes with their small genomes could sense and integrate. In addition, any wrong decision to degrade or inhibit metabolic enzymes, for example when combinations of nutrients are present or when supply is only briefly inhibited, would impair growth.

Another potential reason why no such regulation has evolved could be related to the observation that the regulation of upper and lower glycolysis/gluconeogenesis and directionality of flux are performed by the metabolite concentrations of FBP and PEP, which are cut off from the rest of metabolism by irreversible reactions. We propose that the rationale for this regulatory architecture is product inhibition, which ensures that this essential part of central carbon metabolism is adequately supplied with metabolites, but also ensures that uncontrolled and potentially toxic accumulation of metabolites does not occur. In fact, because the reactions of upper and lower glycolysis are effectively irreversible, even a slight misbalance in flux between these enzymes and biomass demand would result in uncontrolled accumulation of metabolites and, in the absence of a cellular overflow mechanism, these metabolites would quickly reach toxic concentrations, e.g., via their osmotic activities. As demonstrated by the simulation, the existing regulation of central metabolism successfully resolves this problem.

The regulatory architecture of central metabolism accomplishes efficient regulation of fluxes and metabolite pools in response to diverse external conditions, while avoiding toxic accumulation of internal metabolites and integrating multiple conflicting signals with only two regulatory nodes. Central metabolism is a remarkable example of self‐organization of regulatory networks in biology. It provides an elegant solution to the complex, obligatory problem, posed by the biochemistry of central carbon metabolism. All organisms that need to switch between glycolytic and gluconeogenic flux modes face this problem, and we argue that this explains the striking degree of conservation of the phenomenology of shifts between glycolytic and gluconeogenic conditions that we found in different microbial species, ranging from *E. coli*, *B*. *subtilis*, and even wild‐type strains of the lower eukaryote *S*. *cerevisiae* to the reversed phenotypes in *P. aeruginosa*. Conversely, we believe that the quantitative phenotypes exhibited by microbes in such idealized growth shift experiments in the lab can reveal much about their natural environments, ecology and evolutionary origin.

## Materials and Methods

### Bacterial cultures

Strains used in this paper are wild‐type *Escherichia coli* K‐12 NCM3722 (Soupene *et al*, 2003), *Pseudomonas aeruginosa* PAO1 (Stover *et al*, 2000), and *Pseudomonas putida* NIST0129. The culture medium used in this study is $N^{‐}C^{‐}$ minimal medium (Csonka *et al*, 1994), containing K_2_SO_4_ (1 g), K_2_HPO_4_·3H_2_O (17.7 g), KH_2_PO_4_ (4.7 g), MgSO_4_·7H_2_O (0.1 g), and NaCl (2.5 g) per liter. The medium was supplemented with 20 mM NH_4_Cl, as the nitrogen source, and either of the following carbon sources: 20 mM glucose‐6‐phosphate, 20 mM gluconate, 0.2% glucose, 20 mM succinate, 20 mM acetate, 20 mM citrate, 20 mM malate, or 20 mM fumarate.

Growth was then carried out at 37°C in a water bath shaker at 200 rpm, in silicate glass tubes (Fisher Scientific) closed with plastic caps (Kim Kap). Cultures spent at least 10 doublings in exponential growth in pre‐shift medium. For growth shifts, cultured were transferred to a filter paper and washed twice with pre‐warmed post‐shift medium. Cells were resuspended from the filter paper in post‐shift medium and subsequently diluted to an OD of about 0.05.

### Preparation of metabolite samples

Each metabolite sample was filtered, and the filter was immediately plunged in 4 ml ice‐cold methanol (40%)+acetonitrile (40%)+water (20%) and kept in 50‐ml tube. Bacteria were washed off from the filter by pipetting, and the solution was transferred to 15‐ml tube. Original 50‐ml tube was further washed with 4 ml of ice‐cold methanol+acetonitrile+water mix and added to respective 15‐ml tube (total 8 ml). Each sample was dried by Speed Vac, and dried extracts were sent for Mass spec analysis.

### Quantification of intracellular metabolite levels

The dried metabolite extracts were resuspended in 150 µl Milli‐Q water, centrifuged at 4°C, 10,000 rpm for 10 min, and 100 µl precipitate‐free supernatant was transferred to a master 96‐well plate. 25 µl of the master plate was transferred to a 96‐well plate for acquisition, of which 10 µl was injected into a Waters Acquity ultraperformance liquid chromatography (UPLC) system (Waters) with a Waters Acquity T3 column coupled to a Thermo TSQ Quantum Ultra triple quadrupole instrument (Thermo Fisher Scientific) as described previously (Buescher *et al*, 2010). Compound separation was achieved using a gradient of two mobile phases: A, 10 mM tributylamine (ion‐pairing agent), 15 mM acetate, and 5% (*v*/*v*) methanol in water; and B, 2‐propanol. Data were acquired in negative ionization mode using previously published MRM settings (Buescher *et al*, 2010). Peak integration was performed using an in‐house software based on MatLab. A dilution series of standards was used to calculate the concentrations of metabolites in the samples. The final intracellular concentration was calculated from the sample concentration and the extracted intracellular volume.

### Theoretical modeling

The integrated minimal model of metabolism and growth was implemented in MATLAB using the SimBiology toolbox and is described in detail in the Appendix.

## Author contributions

**Severin Josef Schink:** Conceptualization; Formal analysis; Investigation; Writing – original draft; Writing – review and editing. **Dimitris Christodoulou:** Conceptualization; Investigation; Methodology; Writing – review and editing. **Avik Mukherjee:** Conceptualization; Investigation; Methodology; Writing – review and editing. **Edward Athaide:** Investigation. **Viktoria Brunner:** Investigation; Methodology. **Tobias Fuhrer:** Resources; Software; Methodology; Writing – review and editing. **Gary Andrew Bradshaw:** Resources; Methodology. **Uwe Sauer:** Conceptualization; Resources; Funding acquisition; Methodology; Project administration; Writing – review and editing. **Markus Basan:** Conceptualization; Resources; Supervision; Investigation; Writing – original draft; Project administration; Writing – review and editing.

In addition to the CRediT author contributions listed above, the contributions in detail are SJS, DC, AM, TF, US, and MB contributed to the design of the project and writing the manuscript. SJS, DC, EA, and MB performed modeling. AM and MB performed growth experiments. VB, TF, and GAB performed metabolomic measurements.

## Supporting information



Appendix

Expanded View Figures PDF

## Data Availability

The datasets and computer code produced in this study are available in the following databases:
The MATLAB implementation of the model and the data of all figures are available on GitHub: https://github.com/Severin‐schink/Glycolysis‐gluconeogenesis‐switches
Metabolomic data are available on MetaboLights: www.ebi.ac.uk/metabolights/MTBLS3887
