# Supplementary material for: Glycolysis/gluconeogenesis specialization in microbes is driven by biochemical constraints of flux sensing
Source: Mol Syst Biol. 2022 Jan 7;18(1):e10704. doi: 10.15252/msb.202110704 (PMC8738977; doi:10.15252/msb.202110704)
Supplement: Supplementary file 2 — Expanded View Figures PDF [file MSB-18-e10704-s002.pdf]

Expanded View Figures

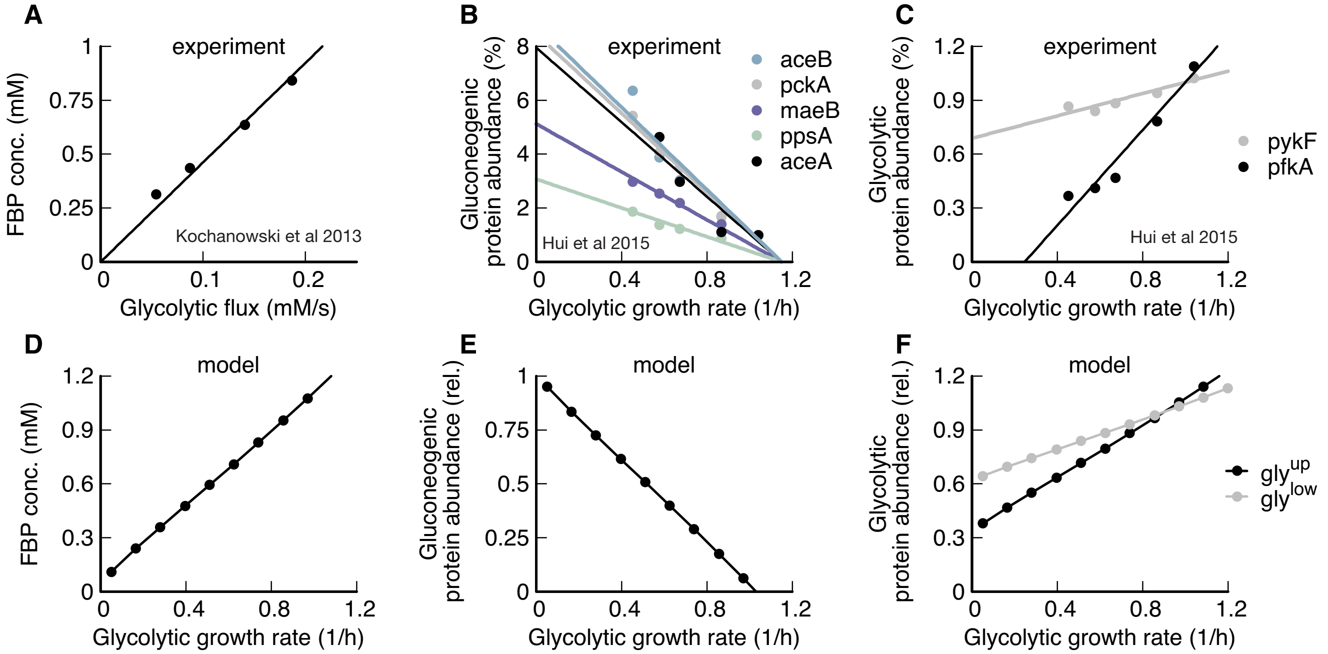

Figure EV1. Metabolic state depends on growth rate.

A During glycolytic growth, FBP linearly increases with growth rate (Kochanowski *et al*, 2013).
B Gluconeogenic enzymes decrease linearly with glycolytic growth rate (Hui *et al*, 2015).
C Glycolytic enzymes increase linearly with glycolytic growth rate (Hui *et al*, 2015).
D–F Simulation results recapitulate experimental evidence.

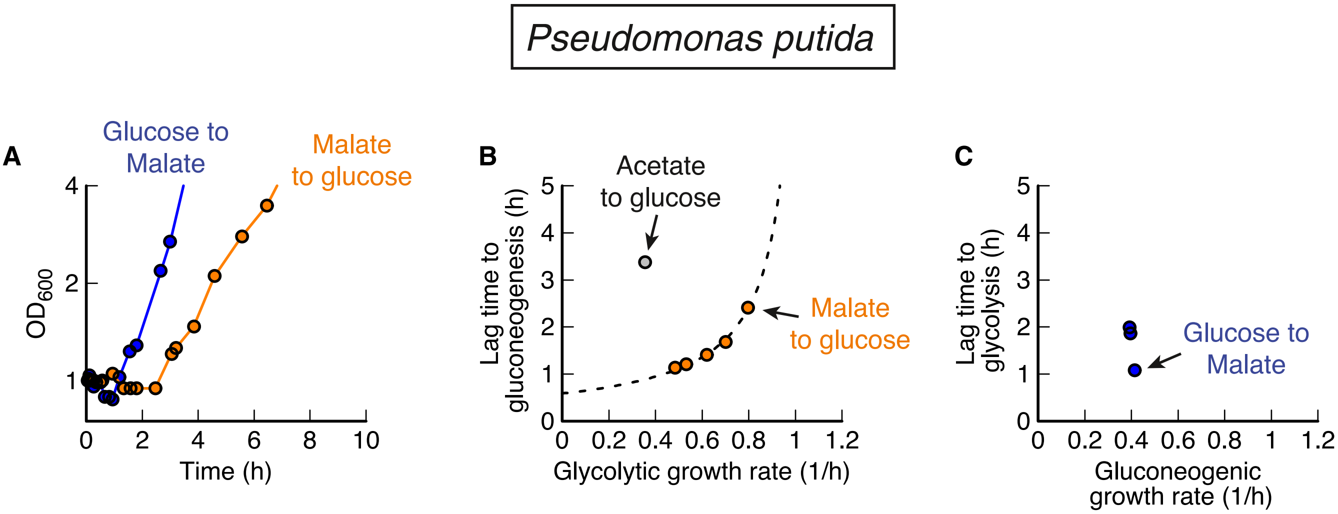

Figure EV2.

◀ **Figure EV2.** ***Pseudomonas putida*** **shifts between glycolytic and gluconeogenic carbon substrates.**

A For shifts between glucose and malate, and vice versa, P. putida shows moderate lag times in both directions.
B Lag times depend on pre-shift growth rate for glycolysis to gluconeogenesis, with acetate to glucose being a clear outlier.
C Lag times for gluconeogenic to glycolytic shifts are of the same magnitude as for the reverse direction. Compared to *P. aeruginosa*, *P. putida* shows longer lags from glucose to malate (0.55 h compared to 1.1 h) and slower growth on the fastest carbon (malate) (1.0/h compared to 0.8/h).

© 2022 The Authors
