# Supplementary material for: Glycolysis/gluconeogenesis specialization in microbes is driven by biochemical constraints of flux sensing
Source: Mol Syst Biol. 2022 Jan 7;18(1):e10704. doi: 10.15252/msb.202110704 (PMC8738977; doi:10.15252/msb.202110704)
Supplement: Supplementary file 3 [file MSB-18-e10704-s003.pdf]

molecular systems biology

Glycolysis/gluconeogenesis specialization in microbes is driven by biochemical constraints of flux sensing

Severin Schink, Dimitris Christodoulou, Avik Mukerjee, Edward Athaide, Viktoria Brunner, Tobias Fuhrer, Gary Bradshaw, Uwe Sauer, and Markus Basan
DOI: 10.15252/msb.202110704

Corresponding author(s): Markus Basan (markus@hms.harvard.edu) , Uwe Sauer (sauer@imsb.biol.ethz.ch)

Review Timeline:

Submission Date:	27th Sep 21
Editorial Decision:	12th Nov 21
Revision Received:	1st Dec 21
Editorial Decision:	9th Dec 21
Revision Received:	10th Dec 21
Accepted:	14th Dec 21

Editor: Maria Polychronidou

Transaction Report:

(Note: With the exception of the correction of typographical or spelling errors that could be a source of ambiguity, letters and reports are not edited. Depending on transfer agreements, referee reports obtained elsewhere may or may not be included in this compilation. Referee reports are anonymous unless the Referee chooses to sign their reports.)

1st Editorial Decision 12th Nov 2021

Thank you again for submitting your work to Molecular Systems Biology. We have now heard back from two of the three referees who agreed to evaluate your study. Unfortunately, after several reminders we have not received a report from reviewer #1. In the interest of time, we have decided to proceed with making a decision based on the two available reports. As you will see below, the reviewers acknowledge that the study is a relevant contribution to the field. They raise however a series of concerns, which we would ask you to address in a revision.

I think that the reviewers' recommendations are clear and therefore there is no need to repeat the points listed below. The most fundamental point was raised by reviewer #2, who points out that experimental validations of novel predictions should be performed to better support the validity of the model and reveal new biology. Reviewer #2 provides specific recommendations in this regard. All issues raised by the reviewers need to be satisfactorily addressed. Please contact me in case you would like to discuss in further detail any of the issues raised.

On a more editorial level, we would ask you to address the following points:

Reviewer #2:

Concerns:

1) While it is interesting that a relatively simple model that combines coarse-grained kinetics and regulation (only six equations in total) can capture key experimental observations such as changes in concentrations of intracellular metabolites FBP and PEP and the length of the lag phase after a carbon source switch, it is important to consider that the model was specifically constructed for this purpose and that the parameters in the model were fitted using experimental data from substrate-switch experiments. So, it should not be surprising that simulations are in fairly good agreement with experimental observations. I would argue that the majority of simulation results that are presented in this work are not independent model predictions, but rather reflect the fact that the authors did a good job fitting the model parameters. In my view, the most interesting (and truly independent) predictions of the model are regarding the activity of metabolic futile cycles. Unfortunately, none of the predictions regarding futile cycling were experimentally tested. It should be feasible to test at least some of the futile cycling predictions using the experimental methods described in Nature chemical biology, 12(7), 482-489, 2016.

2) The authors mention that they ignored the Entner-Doudoroff pathway in their simplified E. coli model. This is probably fine, since it is known (e.g. from 13C-flux studies) that the ED pathway is not very active in E. coli. However, the authors do not mention that they also ignored the pentose phosphate pathway, which is highly active in E. coli, e.g. 13C-flux studies typically show that around 30-40% of glucose is metabolized via the pentose phosphate pathway. The authors should discuss what is the expected impact of ignoring the PPP pathway on their model simulation. For example, by formally considering the PPP pathway, a significant fraction of glucose carbon would be by-passing the FBP node, i.e. since about half of the carbon flowing through PPP will end up producing GAP, and this will likely change the form of the equilibrium equation 7 (which will no longer be quadratic).

3) The authors also suggest that their model predicts that there is a trade-off between the length of the lag-phase and growth rate of cells. Unfortunately, this prediction was also not tested experimentally. One way to evaluate this prediction would be to study E. coli strains that have been adaptively evolved to grow on different carbon sources, e.g. determine the length of lag-phases for strains that have been adaptively evolved to grow on glucose vs. strains that have been adaptively evolved to grow on acetate. What does the model predict that should be expected for such evolved strains? How well do the model predictions match with experimental observations?

Reviewer #3:

The manuscript by Schink and Christodoulou et al. describes a kinetic model of growth shift between glycolysis and gluconeogenesis in bacteria. The aim is to understand the mechanisms determining the duration of lag phases in either direction (glycolysis->gluconeogenesis, gluconeogenesis->glycolysis), and the difference between these two as observed for E. coli. The authors use a simplified central carbon metabolic model by lumping together irreversible reactions and thereby focussing on the key network (pseudo-)nodes - upper and lower glycolysis - and associated (selected) regulatory links. The model is then parameterised using experimental data and used to explore the relationships between metabolite concentrations, protein abundances and flux shifts. The key finding is that the lag time for the shifts have trade-offs with growth rate, energy efficiency and increased lag time for shift in the opposite direction. Metabolomics data from two Pseudomonas sp. is used to demonstrate the trade-off.

Overall, the study provides a new insight into an interesting biochemical problem. While the Results section of the manuscript is generally clear, there are several instances of ambiguity, typos, and impreciseness in Title, Abstract, Introduction and Discussion. Some important biochemical considerations also seem to have been overlooked (details below) and the manuscript would therefore benefit from a thorough revision.

1. Title: the title is misleading as, a) substrate specialization has a very different meaning in ecology / biochemistry (preferential/exclusive utilization of a few substrates from a range of those simultaneously available in the environment) than what the authors study (i.e., switching to by-product utilization after using the primary substrate); b) "flux sensing" is neither evident in the presented data nor adding to the presented concepts, and thus I strongly recommend to avoid using this term.

2. Abstract, Discussion: "....turning the cell 'blind' to...". In my opinion, this is a shallow metaphor that unnecessarily adds to the imprecise anthropomorphising of bacterial physiology. It is shallow because the model does not account for extracellular sensing and several other cellular mechanisms that respond to nutrient and metabolic changes.

Abstract:
3. "very different ways" -> fuzzy

4. "different bacteria" -? How many, which?

5. "integrates...regulation..." -> regulation is too generic and broad a term to describe what is modelled in this study (a few links).

Introduction:

6. Line 31: 'quick' and 'fast' are synonyms, so the grammatically strange construction

7. Line 33: " in the wild": references?

8. "largely elusive"-> what exactly has been elusive? There are several researchers who would claim that it is clear

9. Line 41: "vital" is subjective and imprecise.

10. Line 46: references?

11. Line 54: why "naturally"? In nature, it is often that some other species would utilize the by-products of primary fermentation.

12. Line 71: "Why are..." -> the question is ill phrased as the model cannot show "why" but rather "how".

13. Line 103: "sense fluxes" -> what is meant by this? Is there really a physical way to directly sense fluxes that is applicable here? Metabolite level as a proxy for flux is a consequence of biochemical configuration and not sensing by the cell.

14. Lines 107-8: "...in the absence of information" is inaccurate as no external sensing or other cellular mechanisms (e.g., translation response to low substrates/co-factors etc.) are accounted for.

15. Last Introduction paragraph - the argument if "could have evolved" is spot on but conflicts with the narrative above on "why" etc.

16. There are several typos/grammatical errors etc. throughout and for the sake of time I will not report them all here - most critical being Figure 5 is referred to as Figure 6 on page 17, and on page 13 end I think 'PEP' is meant in place of 'FBP'?

17. Figures: variance is not shown for the experimental data.

18. I did not find any data/code availability statement. Is the data available in Metabolights? Is the Matlab code available to reproduce the results?

19. The model and the interpretation of the results overlooks the role of transporters and transport efficiency. Thus, statements like on line 263 ("must be") etc. are incorrect and Discussion needs to be carefully revised (or a new model built including transporters).

20. 488: "ATP production at relatively low proteome cost" - not entirely true as it assumes that higher proteome will lead to higher flux, the lack of this is exactly why the modelling is being done.

21. 499: "what about enzyme parameters?

22. 500: could IN THEORY use

23. 526: "regulatory architecture AND PARAMETER SPACE"...

24: "conservation of the phenomenology of shifts.." -> why?? This is not striking at all - what else one can expect?

25. The last concluding sentence "environments, ecology and evolutionary origin" is not supported by the results here.

Response to reviewers

We thank the reviewers for their carefully assessment our work. The reviewers have raised several helpful points that we address below. We believe that the changes improved the quality of the manuscript and we think it is now ready for publication.

Reviewer #2:

In this work, the authors developed a relatively simple coarse-grained kinetic model of central carbon metabolism that is combined with equations for allosteric and transcriptional regulation. The main purpose of this model is to describe the lag time that is observed when bacteria switch substrates from glycolytic substrates (e.g. glucose) to gluconeogenic substrates (e.g. acetate), or vice versa. First, the model was trained using experimental data from previous publications. Then, after performing a wide range of simulations using the model, the authors conclude that there are inherent trade-offs between the length of the lag phase, futile cycling, and growth rates of microbes.

Concerns:

1) While it is interesting that a relatively simple model that combines coarse-grained kinetics and regulation (only six equations in total) can capture key experimental observations such as changes in concentrations of intracellular metabolites FBP and PEP and the length of the lag phase after a carbon source switch, it is important to consider that the model was specifically constructed for this purpose and that the parameters in the model were fitted using experimental data from substrate-switch experiments. So, it should not be surprising that simulations are in fairly good agreement with experimental observations. I would argue that the majority of simulation results that are presented in this work are not independent model predictions, but rather reflect the fact that the authors did a good job fitting the model parameters.

> Yes, the model was designed for the specific purpose to describe the dynamics after nutrient switch. Not every model would be able to do this, and we believe that we added just enough components for the model to be able to describe all phenotypes while at the same time being flexible and general enough to scan through parameters to understand what types of kinetics would in principle be possible. A full model of metabolism (one that contains our equations and more) would certainly be able to do the same, but boiling down the model to its key components allowed us to identify fundamental regulatory principles that might have been extremely difficult to identify in a full model.
>
> To be absolutely transparent and reflect that our model is fit to experimental data, we added the following clarifications. <u>Highlighted text: underscored</u>
>
>> Line 181. “To test whether this simple model could recapitulate steady state glycolytic and gluconeogenic growth conditions, we <u>calibrated</u> it <u>with</u> published metabolite and proteomics data of E. coli, [...]”
>>
>> Figure 1. (legend). (C, D&E) <u>Calibration of model to experimental data</u> (from (Basan et al., 2020)) metabolite concentrations and enzyme abundances <u>relative to the highest concentration or abundance.</u>

We also removed the absolute scale of the metabolite measurements, because absolute quantification based on published data is uncertain (see also comment from reviewer #3). This should further focus the reader's attention to the fact that Fig. 1 is a calibration of the model and to highlight that we want to reproduce the differential changes of abundance and concentrations of enzymes and metabolites in glycolysis and gluconeogenesis.

In my view, the most interesting (and truly independent) predictions of the model are regarding the activity of metabolic futile cycles. Unfortunately, none of the predictions regarding futile cycling were experimentally tested. It should be feasible to test at least some of the futile cycling predictions using the experimental methods described in Nature chemical biology, 12(7), 482-489, 2016.

Futile cycles are key to this work, and we would love to measure them. Unfortunately, they are notoriously hard to measure, in particular the ones at the irreversible reactions Pfk and Pyk that are so important in this paper (even the paper cited by the reviewer did not measure these!). We have thought several years about mass spec labeling strategies how to measure these futile cycles at these nodes, but have concluded that it is not possible. To our knowledge previous studies have only quantified futile cycles at the Ppc node and these strategies do not work at either Pyk of Pfk.

To establish a causal link between futile cycles and lag times (without directly measuring them) we have shown in previous work that overexpression of either of the enzymes Pyk or Pfk that are contributing to futile cycling in *E. coli* during shifts to gluconeogenesis dramatically increased lag times [see Extended Data Figure 8, Basan, M., Honda, T., Christodoulou, D., Hörl, M., Chang, Y.-F., Leoncini, E., Mukherjee, A., Okano, H., Taylor, B.R., Silverman, J.M., et al. (2020). A universal trade-off between growth and lag in fluctuating environments. Nature].

2) The authors mention that they ignored the Entner-Doudoroff pathway in their simplified E. coli model. This is probably fine, since it is known (e.g. from 13C-flux studies) that the ED pathway is not very active in E. coli. However, the authors do not mention that they also ignored the pentose phosphate pathway, which is highly active in E. coli, e.g. 13C-flux studies typically show that around 30-40% of glucose is metabolized via the pentose phosphate pathway. The authors should discuss what is the expected impact of ignoring the PPP pathway on their model simulation. For example, by formally considering the PPP pathway, a significant fraction of glucose carbon would be by-passing the FBP node, i.e. since about half of the carbon flowing through PPP will end up producing GAP, and this will likely change the form of the equilibrium equation 7 (which will no longer be quadratic).

The reviewer is right to point out that the precise mathematical form of the growth rate – lag time relation depends on the equilibrium equation. We note that this is not the primary focus of this work. We consider the obligatory existence of the three tradeoffs as the main finding of this work and this result will be unaffected by an altered equilibrium equation. This is also why the model naturally extends to other organisms.

We also want to point out that the equilibrium relation between FBP and PEP is most important during the middle of lag phases. However, in mid lag phase net fluxes are very small and the equilibrium is mainly determined by thermodynamics. Even if the PPP pathway or other pathways were to distort the equilibrium relation during lag phase, as long as the reversible enzymes connecting FBP and PEP via glycolysis are fast or high abundance, FBP and PEP would stay very close to their equilibrium relation.

During growth, the PPP and ED pathways are of course important for metabolism. During exponential growth of *E. coli* about 29% of flux from G6P goes through PPP pathway and 11% through ED (Gerosa et al., 2015, Cell Systems 1, 270–282, http://dx.doi.org/10.1016/j.cels.2015.09.008.). As the reviewer pointed out, some fraction of the metabolic flux from PPP will go directly to GAP (~6%), while the rest (~15%) is cycled back to F6P. Similarly, for the ED pathway flux will go to GAP and PYR. Overall, about 89% of the flux to GAP stems from F6P (Gerosa et al). Because in our model we do not discriminate how flux arrived at F6P, but only describe how it gets converted from F6P to GAP.

We added a note in the manuscript to reflect these comments. Neglecting these parallel pathways is one of many simplifications of the model, but we do not think that our main findings are affected by any of these simplifications.

> Line 172: While the model in Box 1 was formulated to coarse-grain glycolysis via the Embden-Meyerhof-Parnas (EMP) pathway, the dominant glycolytic pathway of E. coli growing on glucose (Gerosa et al., 2015a), other glycolytic pathways, such as the Entner-Doudoroff (ED) or pentose phosphate pathway (PPP), have a similar topology.
>
> Line 285: The form of Eq. (7) is specific to F6P converting to FBP being the irreversible step of upper glycolysis and can change if pathways such as Entner-Doudoroff (ED) or pentose phosphate pathway (PPP) are dominant.

3) The authors also suggest that their model predicts that there is a trade-off between the length of the lag-phase and growth rate of cells. Unfortunately, this prediction was also not tested experimentally. One way to evaluate this prediction would be to study E. coli strains that have been adaptively evolved to grow on different carbon sources, e.g. determine the length of lag-phases for strains that have been adaptively evolved to grow on glucose vs. strains that have been adaptively evolved to grow on acetate. What does the model predict that should be expected for such evolved strains? How well do the model predictions match with experimental observations?

In our paper we propose a total of 4 trade-offs

1. Between lag time to glycolysis and lag time to gluconeogenesis (Fig. 4A-C)
2. Between growth rate and lag time (Fig. 4D-F)
3. Between futile cycling in glycolysis and lag time to gluconeogenesis (Fig. 4H)
4. Between futile cycling in gluconeogenesis and lag time to glycolysis (Fig. 4I)

Because these trade-offs are based on the same six biochemical reaction it is nearly impossible to disentangle them experimentally, but for some this is actually possible.

The reviewer is right that evolution experiments would be one way to test these trade-offs. Evolution experiments tend to be difficult, expensive and very time consuming. To be insightful such experiments must be repeated many times, spanning many different evolution conditions, e.g. different intervals between growth and switching to really map out a Pareto front and establish these tradeoffs. There are not many works in the literature that pass this bar and have actually managed to firmly establish tradeoffs.

The experiments in the literature that is closest to what this would require is Lenski's long term evolution experiment. We actually think that the co-existence of glucose and acetate specialists

in the Lenski experiment can be explained by the tradeoffs outlined in this work (fast growing glucose specialist with long lag time to acetate and a slower growing acetate specialist with short lag to acetate naturally), but a detailed analysis of these evolution experiments would be necessary and is beyond the scope of this work.

But even in an evolution experiment as expansive as Lenski's, the resulting strain is unlikely to be optimized for growth and switching across different growth rates. The required evolution experiment would therefore need not only flipping substrate preference, but also adaptation on diverse growth conditions, in order to show that the growth rate vs lag time relation reappears. 'Simply' evolving one strain for growth on acetate and measuring a lag phase to glucose would not be very conclusive.

So rather than doing an evolution experiment, we asked how evolution has solved this problem in the past. We found that *Pseudomonas aeruginosa* preferentially uses gluconeogenic substrates (and even grows faster than E. coli on glucose!), which gives us an ideal strain that is evolved for gluconeogenic substrates. In addition, we use the fact that bacteria grow at different rates on different substrates, presumably because they have evolved differently on these substrates. Both *E. coli* (Fig. 6D) and *Pseudomonas aeruginosa* (Fig. 6F) show increasing lag times with growth rate. We also found that another species, *Pseudomonas putida,* shows moderate growth and lag times in both direction (Fig. S8). Even for *P. putida* lag time still increases with growth rate. The fact that this relation exists for all species we tested, despite their different evolutionary adaptation is remarkable, and we think it is a strong confirmation that the trade-off between growth rate and lag time exists.

Finally, we want to address the last question of the reviewer, the hypothetical 'what would we expect for evolved strains and how well would model predictions match experimental observations'? Any evolution experiment will lead to a number of mutations. If we evolve for fast growth on acetate, we will not only see changes in metabolic enzymes, but also changes in the rest of the proteome. For example, expression of non-metabolic and non-biosynthetic proteins will likely decrease, because they decrease both growth rate and lag time (e.g. Balakrishnan et al (2021) https://doi.org/10.1101/2021.04.28.441780). Because of these global changes, that are unrelated to the trade-offs we describe, we would need to fit a new parameter set to the evolved strains. In addition, we expect that unless we evolve for a really long time and in multiple conditions, that growth rate and adaptation are not optimal in such an evolved strain. In fact, from our parameter screen (Fig. 5) we know that most parameter combinations are actually pretty bad in most conditions.

Reviewer #3:

The manuscript by Schink and Christodoulou et al. describes a kinetic model of growth shift between glycolysis and gluconeogenesis in bacteria. The aim is to understand the mechanisms determining the duration of lag phases in either direction (glycolysis->gluconeogenesis, gluconeogenesis->glycolysis), and the difference between these two as observed for E. coli. The authors use a simplified central carbon metabolic model by lumping together irreversible reactions and thereby focussing on the key network (pseudo-)nodes - upper and lower glycolysis - and associated (selected) regulatory links. The model is then parameterised using experimental data and used to explore the relationships between metabolite concentrations, protein abundances and flux shifts. The key finding is that the lag time for the shifts

have trade-offs with growth rate, energy efficiency and increased lag time for shift in the opposite direction. Metabolomics data from two Pseudomonas sp. is used to demonstrate the trade-off.

Overall, the study provides a new insight into an interesting biochemical problem. While the Results section of the manuscript is generally clear, there are several instances of ambiguity, typos, and impreciseness in Title, Abstract, Introduction and Discussion. Some important biochemical considerations also seem to have been overlooked (details below) and the manuscript would therefore benefit from a thorough revision.

We thank the reviewer for these helpful suggestions for clarifying our writing and statements.

1. Title: the title is misleading as, a) substrate specialization has a very different meaning in ecology / biochemistry (preferential/exclusive utilization of a few substrates from a range of those simultaneously available in the environment) than what the authors study (i.e., switching to by-product utilization after using the primary substrate); b) "flux sensing" is neither evident in the presented data nor adding to the presented concepts, and thus I strongly recommend to avoid using this term.

a) We thank the reviewer for this good Point. We have adjusted the title to clarify the focus of this work.

b) In the metabolic network that we model, flux direction is indeed sensed by measuring concentrations of key metabolites. This is one of the major points of our work and we think the theoretical model clearly shows this. Moreover, during steady-state, as shown in Fig. S1, our model precisely recapitulates the flux sensor role of FBP that has been experimentally established in previous works [Kochanowski, K., Volkmer, B., Gerosa, L., Haverkorn van Rijsewijk, B.R., Schmidt, A., and Heinemann, M. (2013b). Functioning of a metabolic flux sensor in Escherichia coli. Proceedings of the National Academy of Sciences of the United States of America 110, 1130–1135]. If there is no net flux, metabolism is in equilibrium the concentrations of metabolites are directly related and no differential regulation is possible. This is what we mean with the ‘biochemical constraint in flux sensing’. Therefore, we think that flux-sensing is the appropriate term.

We changed the title to: Glycolysis/gluconeogenesis specialization in microbes is driven by biochemical constraints of ~~dynamic~~ flux sensing. (underscored reflect changes)

2. Abstract, Discussion: "....turning the cell 'blind' to...". In my opinion, this is a shallow metaphor that unnecessarily adds to the imprecise anthropomorphising of bacterial physiology. It is shallow because the model does not account for extracellular sensing and several other cellular mechanisms that respond to nutrient and metabolic changes.

Good point, we changed it to: “[…] rendering the cell unable to sense […]”

Abstract:
3. "very different ways" -> fuzzy

We reworded this sentence to :

Central carbon metabolism is highly conserved across microbial species, but can catalyze very different pathways depending on

4. "different bacteria" -? How many, which?

Changed to:

"Here, we study the dynamic re-organization of central metabolism after switches between the two major opposing pathway configurations of central carbon metabolism, glycolysis and gluconeogenesis in *Escherichia coli, Pseudomonas aeruginosa and Pseudomonas putida*."

These two species is where the majority of the focus is on in this work. We also used *P. putida* see Fig. S8.

5. "integrates...regulation..." -> regulation is too generic and broad a term to describe what is modelled in this study (a few links).

Allosteric interactions and transcription are well known elements of regulation of gene expression and enzyme activity. Even if our model is simplified, it does include regulation. We start the sentence with 'coarse-grained', so it should be clear to the reader that we are not including all known regulations in the model.

Introduction:

6. Line 31: 'quick' and 'fast' are synonyms, so the grammatically strange construction

We have changed this formulation. Please see below (point 7).

7. Line 33: " in the wild": references?

We have included new references and restructured the sentence to:

"Whether in nature, microbiomes or infections, microbes frequently encounter changing environments (Hardcastle and Mann, 1968; Fenchel, 2002; Stocker, 2012; Battin et al., 2016; Forsyth et al., 2018) and their ability to adapt quickly is a key determinants of fitness."

8. "largely elusive"-> what exactly has been elusive? There are several researchers who would claim that it is clearJ

We have clarified this sentence:

"But in comparison with steady state exponential growth, understanding of the physiology of growth transitions, in particular what sets the time-scales of adaptation, has remained largely elusive."

9. Line 41: "vital" is subjective and imprecise.

We have changed the wording to ‘kinetic’ to clarify that the cited works focused on getting kinetic parameters from their modelling:

> “Such metabolic models were successfully expanded to dynamic environments (Zampar et al., 2013; Chassagnole et al., 2002; Chakrabarti et al., 2013; Saa and Nielsen, 2015; Andreozzi et al., 2016; Yang et al., 2019) and used to gather kinetic information about metabolism [...]”

10. Line 46: references?

We have removed this sentence.

11. Line 54: why "naturally"? In nature, it is often that some other species would utilize the by-products of primary fermentation.

Not every microbe lives in a microbial community, and in not all community fermentation products are all used up before the primary product runs out.

We added a qualifier:

> “This naturally leads to bi-phasic growth, if no other microbe is around to utilize this bi-product, where initial utilization of glucose is followed by a switch to acetate.”

12. Line 71: "Why are..." -> the question is ill phrased as the model cannot show "why" but rather "how".

This is a matter of taste. For us, we want to address why-questions in biology even if it is very difficult to obtain definitive answers. The question we are trying to address is: “Why are microorganisms not able to overcome long lag phases using the existing allosteric and transcriptional regulation?” The answer is that trade-offs between lag times, growth rates and futile cycling prevent microbes to optimize all traits simultaneously

13. Line 103: "sense fluxes" -> what is meant by this? Is there really a physical way to directly sense fluxes that is applicable here? Metabolite level as a proxy for flux is a consequence of biochemical configuration and not sensing by the cell.

The term flux sensing is established in the field (e.g., Kochanowski et al https://doi.org/10.1073/pnas.1202582110) and we are using the term is in line with the previous literature. The network described by our model detects both the direction and the magnitude of flux and modulates enzyme activity and enzyme expression levels accordingly (see Fig. S1). For all practical purposes and in linne with previous works we call this kind of regulation “flux sensing”. We think it would actually lead to confusion if we changed the term.

There are potentially many different mechanisms how cells can measure flux. The mechanism in our model is that metabolites can be used as a proxy for cells to sense flux. The metabolite FBP has been experimentally shown to reflect glycolytic flux and directly affect expression of dozens of genes.

14. Lines 107-8: "...in the absence of information" is inaccurate as no external sensing or other cellular mechanisms (e.g., translation response to low substrates/co-factors etc.) are accounted for.

We changed the text to:

> "This choice of direction at low metabolite concentrations becomes the 'default state' of central metabolism and determines the substrate preference."

15. Last Introduction paragraph - the argument if "could have evolved" is spot on but conflicts with the narrative above on "why" etc.

The point we are trying to get across is that glycolysis-specialists *or* gluconeogenesis-specialists could evolve. But why not both? Why are there no bacteria that can grow fast and switch fast on/to all substrates?

We believe that the trade-offs that we describe in our work give some good reasons for that. In the discussion section, we pick up this point again and discuss what kind of problems different regulatory architectures would have, and why we think that the trade-offs that we describe are really uncircumventable.

16. There are several typos/grammatical errors etc. throughout and for the sake of time I will not report them all here - most critical being Figure 5 is referred to as Figure 6 on page 17, and on page 13 end I think 'PEP' is meant in place of 'FBP'?

Thank you for pointing this out. We have proofread the text to try to root out remaining errors.
- We fixed Fig. 5.
- FBP was correct.

17. Figures: variance is not shown for the experimental data.

Error bars are shown whenever possible. Any new data from experiments performed for this work has either error bars that indicate the variance of the data or all repeats are shown individually. In detail:

- Figure 1. Data from https://doi.org/10.1038/s41586-020-2505-4. Inferred from time-series of previous published data. Error bars are not available.
- Figure 2. Data from https://doi.org/10.1038/s41586-020-2505-4. Data is again a time series with single points shown.
- Figure 3. Data from https://doi.org/10.1038/s41586-020-2505-4. Same as Fig. 2
- Figure 6A. Data from us. Three repeats. Error bars show standard deviation
- Figure 6B-C. Data from us. A single repeat is shown.
- Figure 6 D & F. Data from us. Every data point is a shift similar to Fig. 6B&C.
- Figure 7. Data from us. Time series with three repeats at every time-point.

To be transparent, we added a statement to every figure explaining how we generated data, where it is from and why on some figures error bars are missing. In addition, we removed the absolute scale on FBP and PEP (which we are not using in the model) in Fig. 1D and plotted the data on a relative scale. We this this reflects better that there is uncertainty in the quantitative data, and puts the spotlight on the qualitative changes.

18. I did not find any data/code availability statement. Is the data available in Metabolights? Is the Matlab code available to reproduce the results?

We now included a Data availability statement. We uploaded code to github and metabolomics data to Metabolights.

19. The model and the interpretation of the results overlooks the role of transporters and transport efficiency. Thus, statements like on line 263 ("must be") etc. are incorrect and Discussion needs to be carefully revised (or a new model built including transporters).

We thank the reviewer for raising this potential point of confusion. Transport processes are coarse-grained into the model as direct flux to the substrates of the irreversible reactions, either glycolytic carbons or TCA carbons. If there are insufficient transporters present, they will certainly slow down lag times. But note that even most transport systems of glycolytic systems are repressed in the absence of the carbon, but lag times are still very short (Fig. 1H). Thus, the long lag times we observe must be caused by something other than transport. For that reason, we coarse-grained transport into uptake of glycolytic/TCA carbons and focused on central metabolism as the potential culprit for long lag times instead.

To clarify the text, we added a sentence about transport in lines 151-153 and also added a qualifier statement:

> Line 263: "The absence of transient futile cycling, despite the symmetry of regulation and metabolic reactions, means that, <u>according to the model</u>, it must be the allosteric and transcriptional regulations that 'prime' central metabolism of E. coli for the glycolytic direction."

20. 488: "ATP production at relatively low proteome cost" - not entirely true as it assumes that higher proteome will lead to higher flux, the lack of this is exactly why the modelling is being done.

This statement was meant to say that proteome cost to supply the additional energy required for futile cycling is relatively modest, according to the available estimates for ATP production from glycolysis and the TCA cycle [Basan, M., Hui, S., Okano, H., Zhang, Z., Shen, Y., Williamson, J.R., and Hwa, T. (2015a). Overflow metabolism in Escherichia coli results from efficient proteome allocation. Nature *528*.]. These estimates already included the proteome cost of uptake transporters.

Reviewer is right that only more proteins doesn't mean more ATP. In addition to proteome costs directly associated with ATP production, there is also a contribution from transporters and metabolic enzymes. We adapted the discussion to address this point:

The energetic cost of such a wasteful strategy would be relatively low. Because energy production pathways only constitute a relatively small fraction (around 20% (Basan et al., 2015)) of the total cellular proteome and nutrient uptake even smaller (around 1% for glucose uptake (Schmidt et al., 2016)), the cell could compensate ATP dissipated in futile cycling by increasing nutrient uptake and ATP production at a relatively low proteome cost.

21. 499: "what about enzyme parameters?

The trade-offs are not properties of specific enzyme parameters. We showed that they persist even if we screen a large number of parameters (Fig. 5).

22. 500: could IN THEORY use

We changed the sentence to:

In theory, the cell could use a direct input signal from the carbon substrate to allosterically inhibit or even degrade undesired metabolic enzymes.

23. 526: "regulatory architecture AND PARAMETER SPACE"...

We scanned parameter space for a given regulatory architecture and demonstrated the trade-offs (see Fig. 5). Because the trade-offs are only based on the regulatory architecture, we would like to leave this unchanged.

24: "conservation of the phenomenology of shifts.." -> why?? This is not striking at all - what else one can expect?

There are plenty of alternative outcomes that could in principle occur. There is *a priori* no reason to expect the same phenomenology in totally different metabolic shifts.

For example, it might be that all organisms lag long in the same shifts and show similar growth rates on the same substrates, simply because these substrates are 'good' or 'bad'. But this isn't the case. Different organisms show completely opposite patterns of substrate preference and lag times (see Fig. 6 where we compare *E. coli* to *P. aeruginosa*). This means that inherent quality of nutrients is not responsible for these differences.

We believe that the fact that *E. coli*, *B. subtilis*, as well as *S. cerevisiae* all show long lag times only in shifts to gluconeogenesis, but short lag in the other direction is already striking. Why has none of these diverse organisms been able to overcome the problem if such regulation is easy to evolve? Is it a fundamental biochemical property of gluconeogenesis substrates like acetate that makes it unavoidable for all organisms? The answer is no, because other bacteria have overcome this problem, e.g., *P. aeruginosa* switches almost immediately to gluconeogenesis. But as we show with the model, this comes at the cost of lag phases in the opposite direction and the same trade-off only in the opposite direction. We think that it is remarkable that the *P. aeruginosa* shows precisely the reverse phenomenology of *E. coli*.

The fact that lag times increase with growth rate for *all* organisms, independently of their specialization, is another remarkable phenomenon in our opinion.

25. The last concluding sentence "environments, ecology and evolutionary origin" is not supported by the results here.

We have changed the formulation from ”we argue” to “we believe”. This should clarify that this is our opinion. We think articulating such an opinion is acceptable in a discussion paragraph.

> Conversely, we believe that the quantitative phenotypes exhibited by microbes in such idealized growth shift experiments in the lab can reveal much about their natural environments, ecology and evolutionary origin.

1st Revision - Editorial Decision 9th Dec 2021

Thank you for sending us your revised manuscript. We think that the performed revisions have satisfactorily addressed the issues raised by the reviewers. As such, I am glad to inform you that we can soon formally accept the study for publication, pending some minor editorial issues listed below. We would ask you to address these issues in a minor revision.

2nd Authors' Response to Reviewers 10th Dec 2021

Accepted **14th Dec 2021**

Thank you again for sending us your revised manuscript. We are now satisfied with the modifications made and I am pleased to inform you that your paper has been accepted for publication.

EMBO PRESS

YOU MUST COMPLETE ALL CELLS WITH A PINK BACKGROUND ↓

PLEASE NOTE THAT THIS CHECKLIST WILL BE PUBLISHED ALONGSIDE YOUR PAPER

Corresponding Author Name: Prof. Markus Basan

Journal Submitted to: Molecular Systems Biology
Manuscript Number: MSB-2021-10704

Reporting Checklist For Life Sciences Articles (Rev. June 2017)

This checklist is used to ensure good reporting standards and to improve the reproducibility of published results. These guidelines are consistent with the Principles and Guidelines for Reporting Preclinical Research issued by the NIH in 2014. Please follow the journal's authorship guidelines in preparing your manuscript.

A- Figures
1. Data
The data shown in figures should satisfy the following conditions:

- the data were obtained and processed according to the field's best practice and are presented to reflect the results of the experiments in an accurate and unbiased manner.
- figure panels include only data points, measurements or observations that can be compared to each other in a scientifically meaningful way.
- graphs include clearly labeled error bars for independent experiments and sample sizes. Unless justified, error bars should not be shown for technical replicates.
- if n< 5, the individual data points from each experiment should be plotted and any statistical test employed should be justified
- Source Data should be included to report the data underlying graphs. Please follow the guidelines set out in the author ship guidelines on Data Presentation.

2. Captions

Each figure caption should contain the following information, for each panel where they are relevant:

- a specification of the experimental system investigated (eg cell line, species name).
- the assay(s) and method(s) used to carry out the reported observations and measurements
- an explicit mention of the biological and chemical entity(ies) that are being measured.
- an explicit mention of the biological and chemical entity(ies) that are altered/varied/perturbed in a controlled manner.

- the exact sample size (n) for each experimental group/condition, given as a number, not a range;
- a description of the sample collection allowing the reader to understand whether the samples represent technical or biological replicates (including how many animals, litters, cultures, etc.).
- a statement of how many times the experiment shown was independently replicated in the laboratory.
- definitions of statistical methods and measures:
  - common tests, such as t-test (please specify whether paired vs. unpaired), simple χ2 tests, Wilcoxon and Mann-Whitney tests, can be unambiguously identified by name only, but more complex techniques should be described in the methods section;
  - are tests one-sided or two-sided?
  - are there adjustments for multiple comparisons?
  - exact statistical test results, e.g., P values = x but not P values < x;
  - definition of 'center values' as median or average;
  - definition of error bars as s.d. or s.e.m.

Any descriptions too long for the figure legend should be included in the methods section and/or with the source data.

In the pink boxes below, please ensure that the answers to the following questions are reported in the manuscript itself. Every question should be answered. If the question is not relevant to your research, please write NA (non applicable). We encourage you to include a specific subsection in the methods section for statistics, reagents, animal models and human subjects.

USEFUL LINKS FOR COMPLETING THIS FORM

http://www.antibodypedia.com
http://1degreebio.org
http://www.equator-network.org/reporting-guidelines/improving-bioscience-research-repor

http://grants.nih.gov/grants/olaw/olaw.htm
http://www.mrc.ac.uk/Ourresearch/Ethicsresearchguidance/Useofanimals/index.htm
http://ClinicalTrials.gov
http://www.consort-statement.org
http://www.consort-statement.org/checklists/view/32-consort/66-title
http://www.equator-network.org/reporting-guidelines/reporting-recommendations-for-tumo
http://datadryad.org
http://figshare.com
http://www.ncbi.nlm.nih.gov/gap
http://www.ebi.ac.uk/ega
http://biomodels.net/
http://biomodels.net/miriam/
http://jjj.biochem.sun.ac.za
https://osp.od.nih.gov/biosafety-biosecurity-and-emerging-biotechnology/
http://www.selectagents.gov/

B- Statistics and general methods

	Please fill out these boxes ↓ (Do not worry if you cannot see all your text once you press return)
1.a. How was the sample size chosen to ensure adequate power to detect a pre-specified effect size?	Sample size of all experiments was chosen to be 3.
1.b. For animal studies, include a statement about sample size estimate even if no statistical methods were used.	N/A
2. Describe inclusion/exclusion criteria if samples or animals were excluded from the analysis. Were the criteria pre-established?	No samples were excluded
3. Were any steps taken to minimize the effects of subjective bias when allocating animals/samples to treatment (e.g. randomization procedure)? If yes, please describe.	No.
For animal studies, include a statement about randomization even if no randomization was used.	N/A
4.a. Were any steps taken to minimize the effects of subjective bias during group allocation or/and when assessing results (e.g. blinding of the investigator)? If yes please describe.	No.
4.b. For animal studies, include a statement about blinding even if no blinding was done	N/A
5. For every figure, are statistical tests justified as appropriate?	N/A
Do the data meet the assumptions of the tests (e.g., normal distribution)? Describe any methods used to assess it.	N/A
Is there an estimate of variation within each group of data?	N/A

Is the variance similar between the groups that are being statistically compared?	N/A

C- Reagents

6. To show that antibodies were profiled for use in the system under study (assay and species), provide a citation, catalog number and/or clone number, supplementary information or reference to an antibody validation profile. e.g., Antibodypedia (see link list at top right), 1DegreeBio (see link list at top right).	N/A
7. Identify the source of cell lines and report if they were recently authenticated (e.g., by STR profiling) and tested for mycoplasma contamination.	N/A

* for all hyperlinks, please see the table at the top right of the document

D- Animal Models

8. Report species, strain, gender, age of animals and genetic modification status where applicable. Please detail housing and husbandry conditions and the source of animals.	N/A
9. For experiments involving live vertebrates, include a statement of compliance with ethical regulations and identify the committee(s) approving the experiments.	N/A
10. We recommend consulting the ARRIVE guidelines (see link list at top right) (PLoS Biol. 8(6), e1000412, 2010) to ensure that other relevant aspects of animal studies are adequately reported. See author guidelines, under 'Reporting Guidelines'. See also: NIH (see link list at top right) and MRC (see link list at top right) recommendations. Please confirm compliance.	N/A

E- Human Subjects

11. Identify the committee(s) approving the study protocol.	N/A
12. Include a statement confirming that informed consent was obtained from all subjects and that the experiments conformed to the principles set out in the WMA Declaration of Helsinki and the Department of Health and Human Services Belmont Report.	N/A
13. For publication of patient photos, include a statement confirming that consent to publish was obtained.	N/A
14. Report any restrictions on the availability (and/or on the use) of human data or samples.	N/A
15. Report the clinical trial registration number (at ClinicalTrials.gov or equivalent), where applicable.	N/A
16. For phase II and III randomized controlled trials, please refer to the CONSORT flow diagram (see link list at top right) and submit the CONSORT checklist (see link list at top right) with your submission. See author guidelines, under 'Reporting Guidelines'. Please confirm you have submitted this list.	N/A
17. For tumor marker prognostic studies, we recommend that you follow the REMARK reporting guidelines (see link list at top right). See author guidelines, under 'Reporting Guidelines'. Please confirm you have followed these guidelines.	N/A

F- Data Accessibility

18: Provide a "Data Availability" section at the end of the Materials & Methods, listing the accession codes for data generated in this study and deposited in a public database (e.g. RNA-Seq data: Gene Expression Omnibus GSE39462, Proteomics data: PRIDE PXD000208 etc.) Please refer to our author guidelines for 'Data Deposition'.<br><br>Data deposition in a public repository is mandatory for:<br>a. Protein, DNA and RNA sequences<br>b. Macromolecular structures<br>c. Crystallographic data for small molecules<br>d. Functional genomics data<br>e. Proteomics and molecular interactions	Data Availability statement included in manuscript
19. Deposition is strongly recommended for any datasets that are central and integral to the study; please consider the journal's data policy. If no structured public repository exists for a given data type, we encourage the provision of datasets in the manuscript as a Supplementary Document (see author guidelines under 'Expanded View' or in unstructured repositories such as Dryad (see link list at top right) or Figshare (see link list at top right).	Metabolomics data uploaded to Metabolights
20. Access to human clinical and genomic datasets should be provided with as few restrictions as possible while respecting ethical obligations to the patients and relevant medical and legal issues. If practically possible and compatible with the individual consent agreement used in the study, such data should be deposited in one of the major public access-controlled repositories such as dbGAP (see link list at top right) or EGA (see link list at top right).	N/A
21. Computational models that are central and integral to a study should be shared without restrictions and provided in a machine-readable form. The relevant accession numbers or links should be provided. When possible, standardized format (SBML, CellML) should be used instead of scripts (e.g. MATLAB). Authors are strongly encouraged to follow the MIRIAM guidelines (see link list at top right) and deposit their model in a public database such as Biomodels (see link list at top right) or JWS Online (see link list at top right). If computer source code is provided with the paper, it should be deposited in a public repository or included in supplementary information.	Our model was written in MATLAB. The code is available on github.

G- Dual use research of concern

22. Could your study fall under dual use research restrictions? Please check biosecurity documents (see link list at top right) and list of select agents and toxins (APHIS/CDC) (see link list at top right). According to our biosecurity guidelines, provide a statement only if it could.	No.
